# Magnetic Separation of Oil Spills from Water Using Cobalt Ferrite Nanoparticles with Fluorocarbon Functionalization

**DOI:** 10.3390/ijms26146562

**Published:** 2025-07-08

**Authors:** Aljoša Košak, Ajra Hadela, Mojca Poberžnik, Aleksandra Lobnik

**Affiliations:** 1 Faculty of Mechanical Engineering, Center for Sensor Technology, University of Maribor, Smetanova 17, 2000 Maribor, Sloveniaaleksandra.lobnik@um.si (A.L.); 2 Institute for Environmental Protection and Sensors (IOS) Ltd., Beloruska 7, 2000 Maribor, Slovenia; ajra.hadela@ios.si (A.H.); mojca.poberznik@ios.si (M.P.);

**Keywords:** cobalt ferrite, spinel, fluorocarbons, oil spill, adsorption, hydrophobicity, oleophobicity, water remediation

## Abstract

In the present study, we synthesized fluorocarbon-coated cobalt ferrite (CoFe_2_O_4_) magnetic nanoparticles using alkoxysilanes such as trimethoxy(3,3,3-trifluoropropyl)silane (TFPTMS), trimethoxy(1H,1H,2H,2H-nonafluorohexyl)silane (NFHTMS), and triethoxy(1H,1H,2H,2H-perfluorodecyl)silane (PFDTES). The synthesized nanoparticles were characterized by various techniques, including X-ray diffractometry (XRD), transmission electron microscopy (TEM/HRTEM/EDXS), Fourier transform infrared spectroscopy (FTIR), specific surface area measurements (BET), and magnetometry (VSM). To understand their surface characteristics, contact angle (CA) measurements were carried out, providing valuable insights into their hydrophobic properties. Among the samples of CoFe_2_O_4_ coated with fluoroalkoxysilanes, those with PFDTES surface coating had the highest water contact angle of 159.2°, indicating their superhydrophobic character. The potential of the prepared fluoroalkoxysilane-coated CoFe_2_O_4_ nanoparticles for the removal of waste low-SAPS synthetic engine oil from a model aqueous solution was evaluated based on three key parameters: adsorption efficiency (%), adsorption capacity (mg/g), and desorption efficiency (%). All synthesized CoFe_2_O_4_ samples coated with fluoroalkoxysilane showed high oil adsorption efficiency, ranging from 87% to 98%. The average oil adsorption capacity for the samples was as follows: F_3_-SiO_2_@CoFe_2_O_4_ (3.1 g of oil/g of adsorbent) > F_9_-SiO_2_@CoFe_2_O_4_ (2.7 g of oil/g of adsorbent) > F_17_-SiO_2_@CoFe_2_O_4_ (1.5 g of oil/g of adsorbent) as a result of increasing oleophobicity with increasing fluorocarbon chain length. The desorption results, which showed 77–97% oil recovery, highlighted the possibility of reusing the adsorbents in multiple adsorption/desorption cycles.

## 1. Introduction

With increased oil extraction, transportation, and industry activities in recent years, the concern for environmental risks due to oil spill accidents and large amounts of industrial oily wastewater has risen [1]. Oil leakages, especially in the marine environment, are a huge threat to the vulnerable environment and animal and human health [2,3]. Oil spilled in water forms a thin layer on the surface of the water, preventing the penetration of light and the exchange of gases with the atmosphere, which destroys the water environment and smothers the plants and animals living in or near it. Long-term exposure showed higher mortality for embryos and larvae of aquatic organisms at concentrations as low as 0.015 mg/L [1,3]. Oily wastewater can also cause pipe corrosion or interfere with water treatment by consuming dissolved oxygen. Additionally, crude oil and similar oily liquids can contain toxic substances, such as volatile organic components with toxic vapors and polycyclic aromatic hydrocarbons, which are harmful due to their persistent nature [2,3]. Therefore, rapid and effective removal of spilled oil from water is crucial.

Although large oil spills into waters receive more attention, about 90% of oil pollution happens daily due to natural leaks or disposals, transportation leakages, road run-offs, and untreated industrial wastewater from food processing, textiles, metal processing, and mining [1]. An example of an extensive oil leak was the 2010 incident in the Gulf of Mexico, where 100 million barrels of oil were leaked, and it was estimated that only 10% of the leaked oil was recovered by mechanical means. This realization motivated intensive research on new oil spill remediation methods and techniques.

Despite the numerous oil spill control and clean-up methods already available, none of the currently used remediation methods are ideal, as each method has advantages and disadvantages. Most of them are expensive and time-consuming, have low efficiency, and cannot completely recover the oil from water, so research is still focused on exploring new strategies for oil spill remediation [3,4,5,6].

Having different methods available is probably a more innovative approach because each oil spill is unique and should be treated suitably according to its specifics. For example, booms and skimmers might be the best methods for large oil spills as the first step to limit the contaminated area, while after the removal of most of the spilled oil, bioremediation with oil-degrading organisms is more suitable to remove the leftover oils [4,5,7].

Among various methods of removing or collecting oils from water, adsorption is the most used, as it is considered one of the most suitable due to its efficiency, economic viability, and eco-friendliness [8]. Additionally, compared to other methods, such as in-situ burning or the use of dispersants, adsorption does not cause secondary pollution [2,4,5].

An ideal adsorbent should have high oil adsorption capacity, the ability to selectively separate oil and water, fast oil adsorption kinetics, and the ability to float on water. It must also be recyclable, environmentally friendly, and reasonably inexpensive [8]. Nowadays, a wide range of oil absorbents are readily available. Traditional adsorption materials, such as activated carbon, zeolites, clay minerals, silica gel, bentonite, perlite, etc., have proven reliable and effective in adsorption processes. Although they usually offer several advantages, some may also have limitations, such as low adsorption capacity, low oil and water selectivity, high production and operating costs, and limited reuse potential [2]. Many natural materials, such as inorganic minerals (e.g., bentonite, zeolite, perlite) or plant fibers (e.g., cotton, jute, hemp) with intrinsic oil absorbency, are abundant, often found as residues, and are biodegradable. Synthetic polymers, notably sponges or other porous scaffolds, also exhibit extremely high adsorption capacities [8,9].

Most recent research takes advantage of known materials that already possess good adsorption properties and improves those properties or adds new ones for even better adsorbent performance [1,2,8]. Such improvement usually involves surface modification to increase specific surface area, porosity, roughness, and hydrophobicity by adding new functional groups or layers [10]. Hydrophobicity can be achieved by creating low-energy surfaces, often by anchoring non-polar functional groups onto the surfaces [1,10]. While hydroxy (–OH), carboxy (–COOH), amino (–NH_2_), sulfate (–OSO_3_H), and similar functional groups increase the surface energy and exhibit a hydrophilic character, fluorocarbons, hydrocarbons, or silicone-based polymers decrease surface energy and show a hydrophobic character on their surfaces [1,8,11,12,13,14]. A well-known fluoropolymer with hydrophobic properties is polytetrafluoroethylene, also known as Teflon^®^.

In addition to modifying conventional materials with different functional groups, there is a growing interest in advanced nanostructured materials, where the dimensionality significantly impacts their physicochemical properties and selective adsorption capacity. One-dimensional (1D) materials, such as carbon nanotubes, metallic nanowires, nanofibers, and nanorods, stand out due to their high length–diameter ratio and the possibility of targeted surface functionalization, which improves their efficiency in trapping contaminants. Their unique morphologies and high surface areas, combined with tunable hydrophobic surface modifications, allow these nanostructured materials to selectively adsorb oil while repelling water, enhancing the efficiency of oil spill remediation [15,16,17,18,19,20,21]. Furthermore, two-dimensional (2D) materials such as graphene, graphitic carbon nitride (g-C_3_N_4_), MXenes, MoS_2_, and Janus structures stand out due to their layered morphology, large specific surface area, controlled interactions with water and oils, and high density of active sites [22,23,24,25,26,27,28,29,30]. By tailoring their surface chemistry to enhance hydrophobicity or amphiphilicity, these 2D materials further improve the selective separation of oil from water. On this basis, magnetic 2D structures such as g-C_3_N_4_@FeNi_3_ or amphiphilic Janus composites have also been developed to enable efficient oil collection under an external magnetic field [31].

Further development of nanomaterials has led to three-dimensional (3D) porous nanostructures that are intrinsically magnetic, such as magnetic nanoparticles, magnetic nanocomposites, magnetic hydrogels, multilayer magnetic stacked materials, or spatially distributed magnetic lattices [5,32,33,34,35,36,37,38,39]. These materials combine high porosity, functional group arrangement, and magnetic responsivity and often include surface modifications to increase hydrophobicity and selectivity, which together enable multi-level adsorption, effective removal of contaminants, and magnetic separation. This approach aligns with our research objectives, where we investigate non-layered 3D magnetic systems with high surface area and unique properties for the selective separation and removal of oil contaminants.

Recently, fluoroalkoxysilanes have been widely used for hydrophobic or superhydrophobic coatings [10,15,16,17,40], including water repellency [11], oil/water separation [41], self-cleaning [42], self-healing [43,44], anti-icing [42,45], anti-biofouling [46], anti-sticking [47], anti-corrosion [48], and even gas-barrier [49] applications. They are cost-effective and can be applied as thin molecular films without sophisticated equipment, using the sol–gel technique, which is known as a simple but effective and relatively inexpensive way of modifying the surface chemistry of materials [1,11,12]. The hydrophobicity of fluorinated surfaces is closely related to the presence of fluorine. Fluorine is a highly electronegative atom with a high ionization energy, and the C-F bond is highly polarized and stable, giving fluorinated materials their hydrophobicity. For example, fluorinated silica nanoparticles exhibit unique properties such as low surface energy, mechanical strength, and good thermal and chemical resistance [50].

It has been documented that fluorinated compounds have been used to modify the surfaces of various materials, which consequently showed improved hydrophobicity and, in most cases, superhydrophobicity [10,51]. Fluorinated compounds have been used to alter polyurethane sponges to obtain superhydrophobic oil adsorbents. Chen et al. [52] modified a polyurethane sponge with a polydopamine/graphene oxide composite and perfluorooctyltriethoxysilane, resulting in superhydrophobicity, conductivity, and flame retardancy of the sponge. Ruan et al. [53] deposited a polydopamine film on the surface of a polyurethane sponge and modified the surface with fluorinated thiol, resulting in sponges with excellent oil adsorption and recovery properties. Several researchers have used fluorinated compounds for the modification of meshes, fabrics, and other porous materials that can be used in membranes for oil–water separation, which was nicely gathered in a review article by Rasouli and coworkers [1].

In addition to surface modifications and novel material designs, an increasingly promising approach involves incorporating magnetic properties into adsorbents to facilitate their recovery and reuse. Recently, magnetic adsorbents have been gaining increasing attention, particularly due to their unique superparamagnetic and single-domain properties, which allow for magnetic guidance and thus easy removal from polluted waters [54,55]. Combining magnetic materials with hydrophobic surfaces allows for the adsorption or separation of oil from water, enabling a fast and easy method for the collection of these oil-loaded materials, often as the final step of the separation process [54,56].

In most cases, using magnetic nanoparticles has proven to be a practical, reliable, fast, inexpensive, and environmentally friendly approach for adsorbing oil from water. Magnetic nanoparticles have unique physicochemical properties due to their high surface area-to-volume ratio. The smaller the nanoparticle size, the higher the exposed surface area-to-volume ratio and the higher their adsorption capacity and removal efficiency. In addition, smaller magnetic nanoparticles have a more substantial magnetization effect for a stronger and faster magnetic response [54].

Through surface modification or functionalization, magnetic nanoparticles gain chemical stability and additional surface properties, making them excellent oil adsorbents with high adsorption capacities, high recyclability, and low cytotoxicity [1].

Among various magnetic nanomaterials, spinel ferrite nanoparticles, such as CoFe_2_O_4_, represent a particularly promising class of functional materials due to their unique combination of physicochemical and magnetic properties. One of the key advantages of CoFe_2_O_4_ is its tunable magnetic behavior, including moderately high coercivity, high remanence, and stable magnetization. These magnetic properties make it highly suitable for applications such as magnetic separation, catalysis, sensing technologies, and biomedical uses like hyperthermia treatment of tumors [57,58]. Furthermore, the magnetic characteristics of CoFe_2_O_4_ can be adjusted by controlling the particle size, doping, or surface functionalization [59]. In addition to its magnetic properties, CoFe_2_O_4_ also has excellent chemical and thermal stability, which allows for its use in harsh environments, such as acidic or basic media and at high temperatures. Its structural robustness and corrosion resistance further enhance its durability under challenging conditions.

Another crucial advantage of ferrite nanoparticles lies in their surface functionalization versatility—different functional groups (e.g., –NH_2_, –COOH, –SH, –OH, fluoroalkyl chains) can be introduced to tailor selectivity, hydrophilicity/hydrophobicity, adsorption capacity, and biocompatibility according to the target application [58,60,61]. However, despite these benefits, spinel ferrites also pose some challenges. Their high surface energy and interparticle interactions (e.g., Van der Waals and magnetic forces) often lead to agglomeration, reducing dispersion stability and activity. This issue is typically mitigated using stabilizers, carrier matrices, or appropriate surface coatings. Additionally, uncoated ferrite nanoparticles may exhibit cytotoxicity or poor biocompatibility, restricting their use in applications involving contact with living systems. Therefore, coatings with inert or biocompatible materials such as silica (SiO_2_), polyethylene glycol (PEG), or polysaccharides are commonly applied [62,63]. Furthermore, the cost of synthesis, particularly when precise morphological control and reproducibility are required, can be higher compared to more conventional adsorbents (e.g., activated carbon or polymer-based materials). Nevertheless, the high efficiency, selectivity, and reusability of spinel ferrites like CoFe_2_O_4_ often justify the investment, especially in high-value or precision applications. This balanced combination of advantages and limitations renders CoFe_2_O_4_-based materials highly promising candidates for continued investigation, particularly when combined with surface functionalization strategies that enable precise control of interactions with oils, metal ions, or organic contaminants.

Furthermore, many scaffolds, such as foams, sponges, meshes, aerogels, filters, fibers, etc., have also been prepared by incorporating magnetic nanoparticles to obtain magnetically responsive adsorbents. However, their adsorption capacities mostly come from the porous structure of the scaffolds [54]. For example, Yin et al. [64] prepared magnetically responsive superhydrophobic melamine sponges by modifying the melamine sponge with magnetic particles and candle wax, while Liu et al. [65] prepared superhydrophobic magnetic polyurethane sponges simply by soaking them in a suspension containing magnetic nanoparticles and fluorosilane (Actyflon-G502).

According to the currently available literature, fluorocarbons have not been previously employed as hydrophobic surface modification agents for magnetic nanoparticles intended for use in oil adsorption applications, most likely due to the unfavorable environmental connotations associated with their use and some of the findings related to their oleophobicity. Nevertheless, fluorocarbons are useful in various applications due to their unique properties, such as high thermal stability, low surface energy, low reactivity, and non-flammability. Fluorocarbons are thus widely used in refrigeration and air-conditioning systems, aerosol sprays (such as deodorants, paints, and insecticides), anesthetic and medical procedures, non-stick cookware, electrical wire and cable insulation, fire extinguishing systems, processes to make fabrics and surfaces resistant to water and staining, such as lubricants, etc.

This background provided a unique opportunity to explore the so-far untapped potential of using fluorocarbons as hydrophobic agents for functionalizing magnetic nanoparticles specifically tailored for oil–water adsorption. The research aimed to confirm some known properties of fluorocarbons while exploring their potential for improving oil–water separation, with a focus on optimizing efficiency and understanding how different chain lengths and compositions of fluorocarbons affect adsorption efficiency and their practical utility in the remediation of water contaminated with waste engine oil.

In this study, we developed highly efficient (super)hydrophobic adsorbents by functionalizing magnetic nanoparticles of cobalt ferrite (CoFe_2_O_4_) with fluoroalkoxysilanes. For this purpose, three different types of fluoroalkoxysilanes were selected: trimethoxy(3,3,3-trifluoropropyl)silane (TFPTMS), trimethoxy(1H,1H,2H,2H-nonafluorohexyl)silane (NFHTMS), and triethoxy(1H,1H,2H,2H-perfluorodecyl)silane (PFDTES). These silanes differ in the length of their fluorocarbon chains and the arrangement of their backbones (−CF_2_−) and end groups (−CF_3_), which allowed us to study their structural effects systematically. By depositing these silanes on the surface of CoFe_2_O_4_ magnetic nanoparticles, we investigated their influence on hydrophobicity, oleophilicity, and overall oil–water separation performance. The functionalized nanoparticles were subjected to extensive characterization and performance tests to assess their potential for practical applications, particularly for the remediation of water contaminated with waste engine oil.

The key scientific contribution of this study is the systematic comparison of the effect of different fluorocarbon chain lengths on the surface properties of magnetic nanoparticles and their efficiency in removing organic pollutants from water. Although fluorocarbon compounds have already been used for the hydrophobic functionalization of various materials, studies combining fluorinated functional groups and magnetic nanoparticles for the adsorption of oily contaminants are rare. Some existing studies report on the preparation of superhydrophobic magnetic systems for oil removal (e.g., [66,67,68]), but these are mostly non-systematic examples with a limited number of fluorinated reagents tested, without detailed analysis of the influence of the chain length or degree of fluorination. To date, it has not been investigated how exactly the chain length, position of functional groups, and degree of fluorination affect the performance of magnetic nanoparticles as adsorbents for petroleum contaminants. Our approach goes beyond existing studies by closely examining three distinct fluoroalkylsilanes (TFPTMS, NFHTMS, and PFDTES) that differ in key chemical parameters, particularly their chain length (structural variability) and degree of fluorination. Such systematic analysis allows for understanding how the individual chemical attributes affect hydrophobicity, oleophobicity, and surface energy and, hence, adsorption capacity. Furthermore, for the first time, we applied fluorocarbon functionalization on magnetic CoFe_2_O_4_ nanoparticles to obtain simultaneously magnetically responsive and optimized hydrophobic adsorbents to remove oily contaminants efficiently. This synthesis combines the advantages of magnetic nanoparticles (easy separation by magnet) with the specific surface properties afforded by the fluorocarbon functional groups. This results in improved selectivity, adsorption rate, and reusability of the material. Our approach, therefore, represents a significant step forward in developing efficient, sustainable, and practical solutions for water remediation, going beyond previously published work in this field.

## 2. Results and Discussion

### 2.1. Synthesis and Characterization of Magnetic CoFe_2_O_4_ Nanoparticles

**Figure 1:**
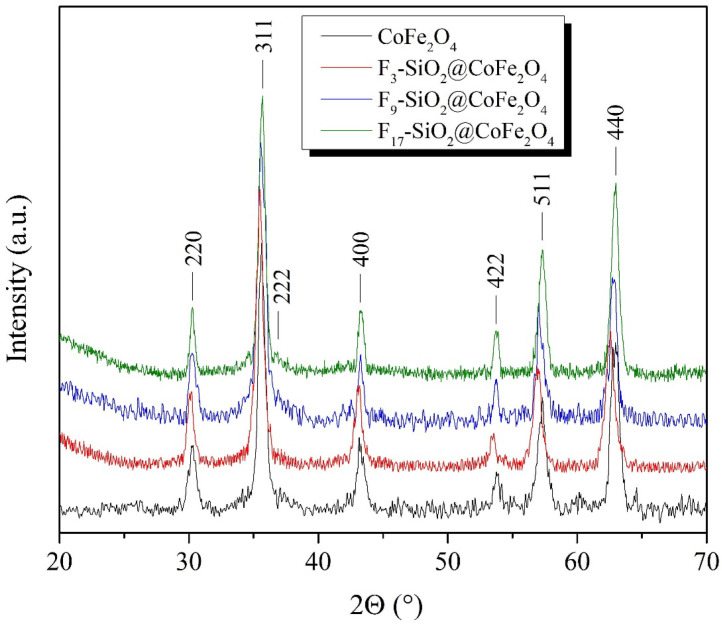
XRD pattern of non-functionalized and functionalized CoFe_2_O_4_ nanoparticles.

Magnetic CoFe_2_O_4_ nanoparticles synthesized by the coprecipitation technique were characterized by X-ray diffractometry (XRD), as shown in Figure 1. The diffraction peaks observed in the prepared samples are consistent with the cubic spinel crystal structure (JCPDS card 22-1086). The XRD pattern shows diffraction lines at 2θ values of 30.2°, 35.6°, 37.2°, 43.3°, 53.7°, 57.2°, and 62.8°, which for the CoFe_2_O_4_ sample correspond to the cubic crystalline planes (220), (311), (222), (400), (422), (511), and (440). Using the Debye–Scherrer equation (Equation (1)), the CoFe_2_O_4_ crystallite size was calculated to be 12.5 ± 0.4 nm based on the broadening of the most prominent diffraction peak corresponding to the (311) crystal plane [69,70]:

(1) $$D_{hkl}=\frac{K\cdot \lambda }{\beta_{hkl}\cdot \cos\theta_{B}}$$

where *D*_hkl_ is the crystallite size (nm) in the direction perpendicular to the lattice planes, *K* is a dimensionless numerical shape factor (typically around 0.94), *λ* is the X-ray wavelength (1.5406 Å for Cu*Kα* radiation), *β* is the full-width at half-maximum of the X-ray diffraction peak in radians, and *θ*_B_ (°) is the Bragg angle.

The lattice constant (*a*) of the cubic crystal structure was calculated using the Bragg equation (Equation (2)), based on the positions of the individual diffraction peaks and the corresponding Miller indices (*h k l*):

(2) $$d=\frac{a}{\sqrt{h^{2}+k^{2}+l^{2}}}$$

where *d*d (nm) is the spacing between the crystal planes in the cubic crystal lattice. The crystal lattice parameter (*a*) corresponding to the cubic spinel crystal structure, calculated to be 0.8350 nm based on Bragg’s law, is in good agreement with the JCPDS database (JCPDS 22-1086).

The X-ray density (*ρ*_x_) of the CoFe_2_O_4_ sample was calculated to be 5.35 g/cm^3^ according to Equation (3) [71]:

(3) $$\rho_{x}=\frac{8\cdot M_{W}}{N_{A}\cdot a^{3}}$$

where *a* (m) is the lattice constant, *M*_w_ is the molecular weight (g/mol) of CoFe_2_O_4_, and *N*_A_ is the Avogadro number.

X-ray diffraction analysis of the surface-functionalized F_3_-SiO_2_@CoFe_2_O_4_, F_9_-SiO_2_@CoFe_2_O_4_, and F_17_-SiO_2_@CoFe_2_O_4_ nanoparticles, on the one hand, reveals the position of prominent diffraction peaks that coincide with the position of the CoFe_2_O_4_ diffraction peaks, indicating that the CoFe_2_O_4_ crystal cubic spinel structure remains unchanged even after the functionalization process with TFPTMS, NFHTMS, and PFDTES fluoroalkoxysilanes. On the other hand, the XRD diffractograms of the samples of the surface-functionalized CoFe_2_O_4_ nanoparticles show broad amorphous peaks at 2θ diffraction angles of 20–28° due to the presence of amorphous silicon dioxide (SiO_2_) bound to the CoFe_2_O_4_ spinel core, further confirming a successful coating process.

**Figure 2:**
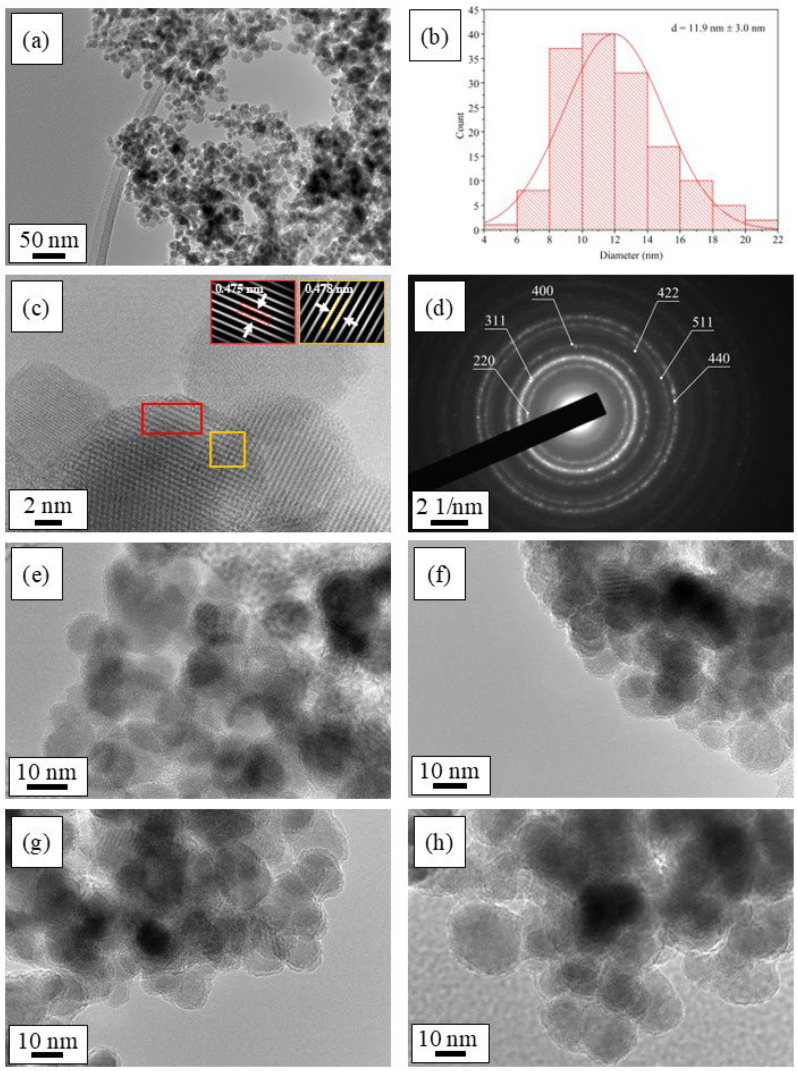
TEM micrographs of (**a**) CoFe_2_O_4_ nanoparticles with (**b**) histogram of the particle size distribution, (**c**) high-resolution image (HRTEM) of CoFe_2_O_4_ nanoparticles with inset showing estimated interplanar spacings (*d*ₕₖₗ) of 0.475 nm and 0.478 nm, indicated by arrows, (**d**) diffraction patterns of CoFe_2_O_4_ nanoparticles, (**e**) SiO_2_@CoFe_2_O_4_ nanoparticles, and (**f**) F_3_-SiO_2_@CoFe_2_O_4_, (**g**) F_9_-SiO_2_@CoFe_2_O_4_, and (**h**) F_17_-SiO_2_@CoFe_2_O_4_ nanoparticles at a molar ratio of P 1.

The transmission electron (TEM) micrographs in Figure 2 represent the morphological properties of the prepared CoFe_2_O_4_ nanoparticles and functionalized F_3_-SiO_2_@CoFe_2_O_4_, F_9_-SiO_2_@CoFe_2_O_4_, and F_17_-SiO_2_@CoFe_2_O_4_ nanoparticle core–shell nanostructures obtained using the TFPTMS, NFHTMS, and PFDTES alkoxysilanes at a molar ratio of P 1.

Figure 2a reveals that the resulting CoFe_2_O_4_ nanoparticles have a relatively spherical morphology with a particle size distribution estimated at (11.9 ± 3.0) nm, as indicated by the histogram in Figure 2b. After coating the magnetic CoFe_2_O_4_ nanoparticles with the TEOS precursor, a thin homogeneous SiO_2_ layer is formed on the surface of the nanoparticles, as seen in Figure 2e. The particle size increases slightly to (12.1 ± 2.5) nm. Comparatively, the CoFe_2_O_4_ magnetic nanoparticles additionally functionalized with TFPTMS, NFHTMS, and PFDTES fluoroalkoxysilanes exhibit slightly larger average sizes due to the surface coating, with particle size distributions of (12.7 ± 3.1) nm for the F_3_-SiO_2_@CoFe_2_O_4_, (14.9 ± 3.7) nm for the F_9_-SiO_2_@CoFe_2_O_4_, and (18.6 ± 5.4) nm for the F_17_-SiO_2_@CoFe_2_O_4_ sample.

The average crystallite size of the CoFe_2_O_4_ nanoparticles, calculated from the (311) peak broadening in the XRD pattern using the Scherrer equation (12.5 ± 0.4) nm, represents the size of the core crystalline domain within individual nanoparticles and agrees well with the particle size obtained from the TEM analysis (11.9 ± 3.0) nm, confirming that the as-synthesized uncoated nanoparticles are likely monocrystalline.

The observed increase in the size of the functionalized nanoparticles is clearly attributed to the silane surface coating, which forms an additional surface layer around the crystalline core. This strong agreement between the XRD and TEM results validates the crystalline core size and highlights the critical influence of the surface functionalization on the overall particle dimensions.

Moreover, the TEM images reveal partial agglomeration in the functionalized samples, consistent with magnetic interactions between nanoparticles. These findings, together with the XRD results, provide a coherent picture of the particle morphology, crystalline domain size, and aggregation behavior. Additional characterization techniques, such as high-resolution imaging and elemental analysis, further contribute to understanding the effects of surface modification.

The high-resolution transmission electron micrograph (HRTEM) of the CoFe_2_O_4_ nanoparticles in Figure 2c reveals the distribution of atoms in the material at the nanoscale. By in-depth analysis of this image using the Fast Fourier Transform (FFT), it was possible to estimate the interplanar spacing, representing the distance between the planes of the crystal lattice in the material structure. The inset of the image (Figure 2c) shows a magnified view of a specific region where interplanar spacings of 0.475 nm and 0.478 nm are typical of the crystal structure of CoFe_2_O_4_. The electron diffraction pattern of the CoFe_2_O_4_ nanoparticles (Figure 2d) shows the crystalline nature of the as-prepared powders, consistent with the HRTEM image of CoFe_2_O_4_. Each concentric diffraction ring in the diffraction pattern belongs to a spinel crystal structure.

**Figure 3:**
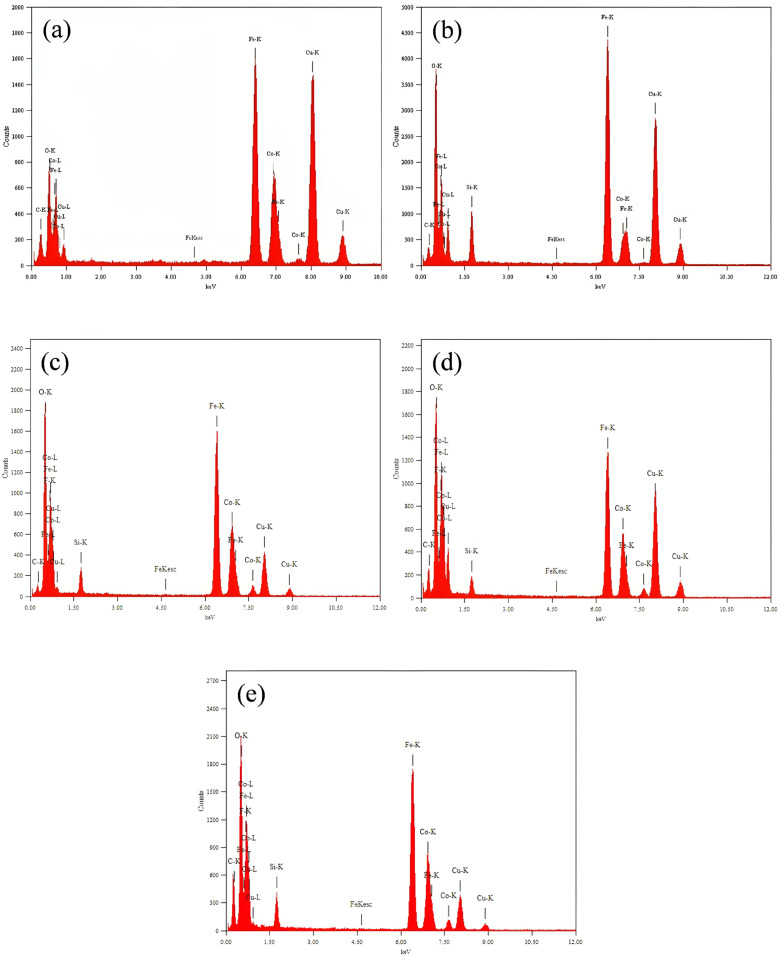
EDXS spectra of (**a**) CoFe_2_O_4_, (**b**) SiO_2_@CoFe_2_O_4_, (**c**) F_3_-SiO_2_@CoFe_2_O_4_, (**d**) F_9_-SiO_2_@CoFe_2_O_4_, and (**e**) F_17_-SiO_2_@CoFe_2_O_4_ nanoparticles.

The energy dispersive X-ray spectroscopy (EDXS) pattern of the CoFe_2_O_4_ nanoparticles, shown in Figure 3, demonstrates the existence of cobalt (Co), iron (Fe), and oxygen (O), implying the successful formation of CoFe_2_O_4_ nanostructures. EDXS analysis revealed that the synthesized cobalt ferrite particles consist of 25.8 wt.% Co, 47.7 wt.% Fe, and 26.5 wt.% O. These values are very close to the theoretical stoichiometric composition of CoFe_2_O_4_ (25.1 wt.% Co, 47.6 wt.% Fe, and 27.3 wt.% O). The calculated atomic ratios based on the measured mass fractions give a Co:Fe:O ratio of approximately 1:1.95:3.78, which is in a good agreement with the ideal stoichiometric ratio of 1:2:4. The minor deviations may be due to inherent limitations of the EDS technique, mainly due to imperfect oxygen detection and possible surface contamination, but the results clearly indicate a near-ideal stoichiometric composition of the cobalt ferrite. At the same time, the spectrum of SiO_2_@CoFe_2_O_4_ additionally shows the expected peak of silicon (Si) due to the presence of the SiO_2_ coating.

The EDXS spectra of the F_3_-SiO_2_@CoFe_2_O_4_, F_9_-SiO_2_@CoFe_2_O_4_, and F_17_-SiO_2_@CoFe_2_O_4_ nanoparticles, as shown in Figure 3, confirm the presence of carbon (C), oxygen (O), fluorine (F), cobalt (Co), iron (Fe), and silicon (Si). This proves the successful surface functionalization of the CoFe_2_O_4_ nanoparticles with the fluoroalkoxysilanes TFPTMS, NFHTMS, and PFDTES, resulting in the formation of the F_3_-SiO_2_@CoFe_2_O_4_, F_9_-SiO_2_@CoFe_2_O_4_, and F_17_-SiO_2_@CoFe_2_O_4_ core–shell nanostructures. It is worth noting that the low fraction of copper (Cu) and carbon (C) elements can be attributed to the transparent carbon film supported by the transmission electron microscope (TEM) copper lattice.

After close inspection of the visual data shown in Figure 3, it becomes clear that the task of visually determining the presence or absence of fluorine in the F_3_-SiO_2_@CoFe_2_O_4_, F_9_-SiO_2_@CoFe_2_O_4_, and F_17_-SiO_2_@CoFe_2_O_4_ samples is challenging. This complexity is due to the minimal difference in X-ray line energy between F-K (F-Kα 677eV) and Fe-L (Fe-Lα 704 eV), which is only 27 eV. This minimal difference makes it virtually impossible to visually confirm the presence or absence of fluorine in this spectral range with complete confidence. However, traditional energy dispersive X-ray spectroscopy (EDXS) performed on the F_3_-SiO_2_@CoFe_2_O_4_, F_9_-SiO_2_@CoFe_2_O_4_, and F_17_-SiO_2_@CoFe_2_O_4_ samples identified their elemental composition as cobalt (Co), iron (Fe), carbon (C), oxygen (O), silicon (Si), and fluorine (F). The EDX microanalysis showed that these samples contain fluorine in a concentration range of 0.15 wt.% for F_3_-SiO_2_@CoFe_2_O_4_, 0.78 wt.% for F_9_-SiO_2_@CoFe_2_O_4_, and 1.67 wt.% for F_17_-SiO_2_@CoFe_2_O_4_. These values increase gradually from F_3_-SiO_2_@CoFe_2_O_4_ over F_9_-SiO_2_@CoFe_2_O_4_ to F_17_-SiO_2_@CoFe_2_O_4_, directly correlated to the increasing fluorocarbon chain length. However, it should be admitted that EDXS may not be the optimum technique for verifying the presence of fluorocarbons when amorphous carbon is used as a support. This limitation arises mainly from the spectral overlap of the carbon and fluorine peaks in the EDXS spectrum, which may compromise the accuracy of the results, whereas complementing EDXS with other characterization techniques, such as Fourier transform infrared spectroscopy (FTIR), may provide more convincing evidence of the presence of fluorocarbons in the samples.

**Figure 4:**
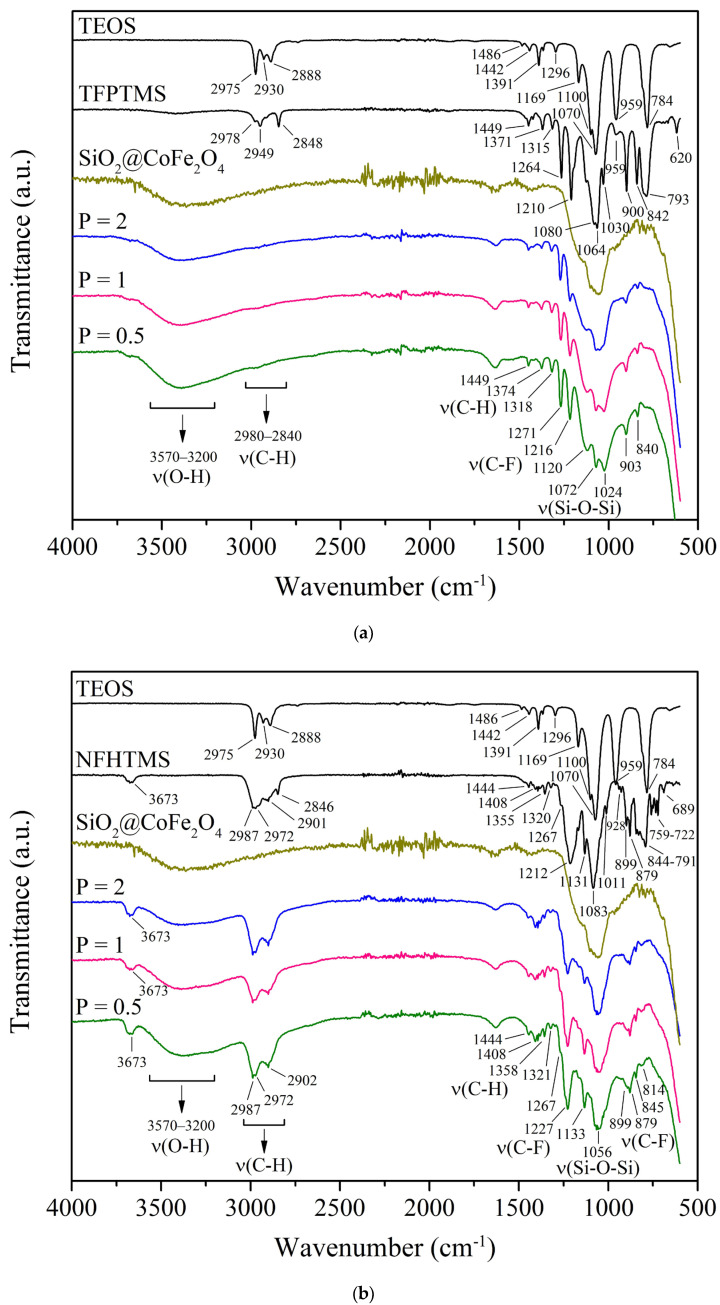

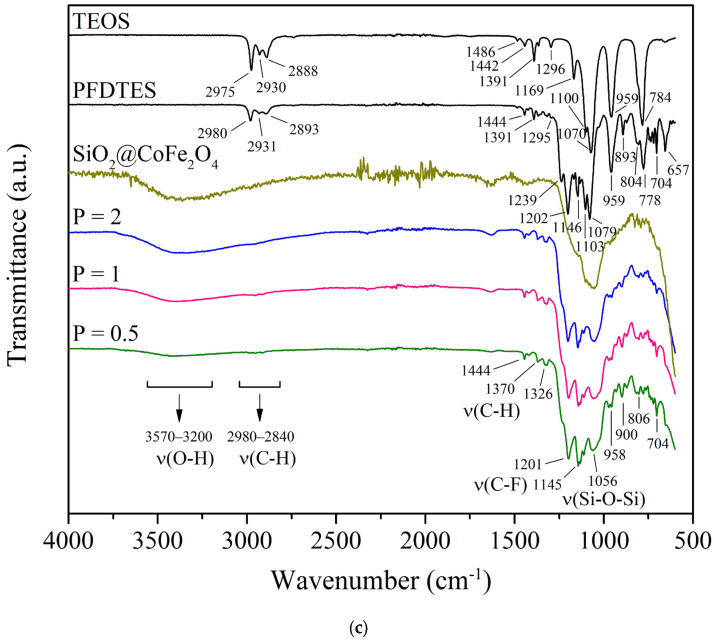
FT-IR spectra of (**a**) F_3_-SiO_2_@CoFe_2_O_4_ nanoparticles, (**b**) F_9_-SiO_2_@CoFe_2_O_4_ NPs, and (**c**) F_17_-SiO_2_@CoFe_2_O_4_, functionalized at molar ratios of P 2, 1, and 0.5.

To verify the binding of fluoroalkoxysilanes to the nanoparticle surface, FTIR spectra of CoFe_2_O_4_ nanoparticles were collected before and after the deposition of fluoroalkoxysilanes, and the results are shown in Figure 4. The characteristic peaks of the silica (SiO_2_) and fluorocarbon coatings were determined, and the spectra of the obtained samples were compared with the spectra of known standards (TEOS, TFPTMS, NFHTMS, and PFDTES) to confirm the presence of the SiO_2_ shell and fluorocarbon chains. FTIR spectra of samples prepared at different molar ratios of P (2, 1, and 0.5) are shown.

The FTIR spectrum of the synthesized SiO_2_@CoFe_2_O_4_ nanoparticles reveals two broad peaks near 3390 cm^−1^ and 1630 cm^−1^, attributable to the free silanol (Si–OH) groups. Despite the typical absorptions of silanol groups in the 950–810 cm^−1^ range, these are not well evident for the SiO_2_@CoFe_2_O_4_ in our spectrum. The presence of one or more strong bands in the range 1110–1000 cm^−1^ can generally be attributed to silicon alkoxy groups (Si–OR), but if siloxane (Si–O–Si) groups are present in the sample, their strong absorption in the same range may obscure the bands attributed to silicon alkoxy groups [72]. Siloxanes typically exhibit pronounced infrared bands within the 1130–1000 cm^−1^ region due to the Si–O–Si stretching vibrations. As the chains of siloxane extend, the absorption of Si–O–Si tends to broaden and increase in complexity, thereby resulting in two or more overlapping bands [72], as shown in Figure 4. As expected, according to the silica (SiO_2_) structure, the presence of a strong and broadband peak at 1070 cm^−1^ for the SiO_2_@CoFe_2_O_4_ nanoparticles in Figure 4 can be assigned to asymmetric stretching vibrations of Si–O–Si bonds. This substantiates the creation of a SiO_2_ shell on the surface of the CoFe_2_O_4_ nanoparticles, as also evident from the TEM images (Figure 2e–h).

The FTIR spectra of the CoFe_2_O_4_ samples coated with fluoroalkoxysilane were then obtained using TFPTMS, NFHTMS, and PFDTES. Peaks in the range from 2980 cm^−1^ to 2890 cm^−1^ are the result of stretching vibrations of C-H bonds in alkyl chains, and peaks in the range of 1455 cm^−1^ to 1310 cm^−1^ and at 720 cm^−1^ are the result of in-plane bending of –CH_3_ and –CH_2_– vibrations. The intensity and position of these bands usually vary slightly due to differences in the chain length of the fluorocarbons, but these variations were not sufficiently distinct in the spectra. Methyl groups usually show two strong and distinct bands, one at 2960 cm^−1^ due to asymmetric stretching and the other at 2870 cm^−1^ due to symmetric stretching, while methylene groups show asymmetric stretching at about 2930 cm^−1^ and symmetric stretching at about 2850 cm^−1^ [73,74].

As shown in Figure 4, all the CoFe_2_O_4_ samples coated with fluoroalkoxysilane have two broad absorption peaks in the range of 3570 cm^−1^ to 3200 cm^−1^ and at 1630 cm^−1^, which can be attributed to free silanol (Si–OH) groups. The prominent absorption peaks appearing between 1070 cm^−1^ and 1020 cm^−1^ are the characteristic Si–O–Si stretching vibrational bands of the fluoroalkoxysilane-coated CoFe_2_O_4_ nanoparticles, like what we previously observed for the SiO_2_@CoFe_2_O_4_ sample.

Moreover, all the fluoroalkoxysilane-coated CoFe_2_O_4_ samples exhibit strong absorption bands between 1270 cm^−1^ and 1100 cm^−1^ corresponding to C–F stretching vibrations, whereas the rocking and wagging vibrations of C–F are typically shown in the range from 746 cm^−1^ to 620 cm^−1^, indicating that the functionalization of the CoFe_2_O_4_ nanoparticles with TFPTMS, NFHTMS, and PFDTES alkoxysilanes was successful [73,75,76].

To complement the structural and morphological insights obtained from the XRD and TEM analyses, Brunauer–Emmett–Teller (BET) surface area measurements were carried out to further assess the specific surface area and the influence of surface functionalization. While XRD and TEM confirmed the crystalline core size and visualized the morphological changes due to the silane coatings, BET analysis quantitatively evaluated the specific surface area, which is affected by the particle size, aggregation state, and surface coverage. These measurements offer an additional dimension to understanding the physicochemical characteristics of the nanoparticles.

BET analysis was performed on the CoFe_2_O_4_ nanoparticles and the functionalized F_3_-SiO_2_@CoFe_2_O_4_, F_9_-SiO_2_@CoFe_2_O_4_, and F_17_-SiO_2_@CoFe_2_O_4_ nanoparticles, prepared at molar ratios of P 2, 1, and 0.5. The BET analysis shows a specific surface area (*S*_A_) of 82.5 m^2^/g for CoFe_2_O_4_. According to the *S*_A_ at a relative pressure (*p*/*p*_0_) of 0.3, the average particle size (*D*_BET_) of the CoFe_2_O_4_ nanoparticles was calculated to be 13.6 nm, assuming the sphericity of the nanoparticles, using the equation *S_A_* = 6/(*D*_BET_∙*ρ*_x_), where *ρ*_x_ is a density of 5.35 g/cm^3^, calculated using Equation (3). The obtained average particle size (*D*_BET_) is slightly larger than the average particle size value of 12.5 nm, obtained from XRD, most probably due to the agglomeration of uncoated CoFe_2_O_4_ nanoparticles.

For the F_3_-SiO_2_@CoFe_2_O_4_ sample, which has the shortest fluoroalkyl chain (TFPTMS), the specific surface area (*S*_A_) shows a slight increase from 94.4 m^2^/g to 101.3 m^2^/g as the P value decreases from 2 to 0.5 (Table 1). This change most probably corresponds to an increase in the surface concentration of the TFPTMS precursor molecules. Consequently, the *D*_BET_ particle size decreases from 11.86 nm to 11.05 nm. This trend is consistent with an inverse relationship between *S*_A_ and particle size: smaller particles have a larger surface area per unit mass, and vice versa. The shorter TFPTMS chains on the surface of the F_3_-SiO_2_@CoFe_2_O_4_ nanoparticles are likely to be more tightly and uniformly packed, as they are less entangled with each other compared to longer chains such as NFHTMS and PFDTES in the F_9_-SiO_2_@CoFe_2_O_4_ and F_17_-SiO_2_@CoFe_2_O_4_ samples, respectively. This may contribute to a more compact and dense coating that prevents nanoparticle aggregation by creating a spatial barrier, which, in turn, helps to maintain a higher *S*_A_ and smaller *D*_BET_, compared to the F_9_-SiO_2_@CoFe_2_O_4_ and F_17_-SiO_2_@CoFe_2_O_4_ samples, as shown in Table 1 [77].

Conversely, the surface functionalization of the CoFe_2_O_4_ nanoparticles with NFHTMS and PFDTES fluoroalkoxysilanes at molar ratios of P 2, 1, and 0.5 results in a significant decrease in *S*_A_, from 92.1 m^2^/g to 70.8 m^2^/g for the F_9_-SiO_2_@CoFe_2_O_4_ nanoparticles and from 44.9 m^2^/g to 0.8 m^2^/g for the F_17_-SiO_2_@CoFe_2_O_4_ nanoparticles, while the molar ratio P decreases from 2 to 0.5. Consequently, the *D*_BET_ nanoparticle size increases from 12.1 nm to 15.8 nm for the F_9_-SiO_2_@CoFe_2_O_4_ sample/, and from 24.9 nm to 1399.3 nm for the F_17_-SiO_2_@CoFe_2_O_4_ sample.

In these two instances, the surface of CoFe_2_O_4_ is covered with long-chain NFHTMS and PFDTES precursor molecules, which, due to the length and the structure of the fluoroalkyl chains, create a physical barrier around each nanoparticle, increasing their hydrodynamic radius compared to bare CoFe_2_O_4_ nanoparticles [78,79]. This contributes to the decreases in *S*_A_ and increases in *D*_BET_ of the F_9_-SiO_2_@CoFe_2_O_4_ and F_17_-SiO_2_@CoFe_2_O_4_ nanoparticles, as seen in Table 2, due to the increase in the concentration of NFHTMS and PFDTES precursor molecules on the surface of the nanoparticles when the molar ratio of P is decreased from 2 to 0.5. Longer chains most probably lead to more pronounced steric hindrance between the chains themselves, and the conformation of the chains, whether they stretch or coil, can also play an important role, leading to less efficient packing on the surface of the nanoparticles [78]. These structural factors affect the specific surface area and other key surface properties of nanoparticles, such as hydrophobicity or hydrophilicity, surface energy, and the distribution of functional groups. Consequently, they significantly determine the nature of the surface interactions of nanoparticles and are directly reflected in their functionality and performance in specific applications, such as oil spill remediation.

**Figure 5:**
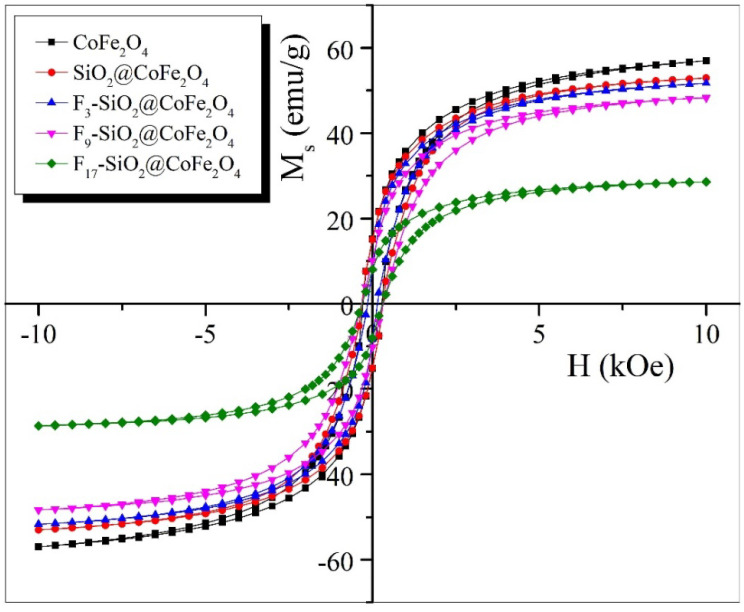
Magnetic characteristics of the CoFe_2_O_4_ (■), SiO_2_@CoFe_2_O_4_ (●), F_3_-SiO_2_@CoFe_2_O_4_ (▲), F_9_-SiO_2_@CoFe_2_O_4_ (▼), and F_17_-SiO_2_@CoFe_2_O_4_ (♦) samples at a molar ratio of P 1.

Figure 5 illustrates the effect of surface modifications on the magnetic behavior of CoFe_2_O_4_ nanoparticles. The graph presents the magnetic hysteresis curves for the uncoated CoFe_2_O_4_ nanoparticles and CoFe_2_O_4_ nanoparticles coated with silica (SiO_2_@CoFe_2_O_4_) and fluoroalkoxysilanes of varying chain lengths (F_3_-SiO_2_@CoFe_2_O_4_, F_9_-SiO_2_@CoFe_2_O_4_, and F_17_-SiO_2_@CoFe_2_O_4_).

Magnetic measurements were performed using VSM at room temperature with a maximum applied field of up to 10 kOe. The samples studied showed characteristic S-shaped hysteresis loops typical of hard magnets with moderate specific magnetizations and relatively high coercivities [80]. From these hysteresis loops, several magnetic parameters were obtained, including the specific magnetization (*M*_s_), remanent magnetization (*M*_r_), and coercivity (*H*_c_), the values of which are summarized in Table 2.

At the maximum magnetic field strength (*H* = 10 kOe), the specific mass magnetization (*M*_s_) of the uncoated CoFe_2_O_4_ nanoparticles reaches a value of 56.9 emu/g during magnetizing, as shown in Figure 5. After removal of the external magnetic field, their magnetic response decreases, and at zero field strength (*H* = 0), they retain a remanent magnetization (*M*_r_) of about 15.2 emu/g.

The shape of the hysteresis curve reveals that the coercivity (*H*_c_) of the CoFe_2_O_4_ nanoparticles is 269.7 Oe, confirming their ferrimagnetic nature. This is due to the pronounced magnetocrystalline anisotropy, which acts as an energy barrier to spin relaxation during the transition from the ferrimagnetic to the superparamagnetic state [81,82].

Such ferrimagnetic nanoparticles pose a challenge for manipulation in solutions, as they tend to aggregate under the influence of an external magnetic field due to interparticle magnetic interactions. In contrast, superparamagnetic nanoparticles, which do not exhibit remanence (*M*_r_) or coercivity (*H*_c_), remain well-dispersed even in the presence of a magnetic field. Encapsulation of ferrimagnetic nanoparticles with non-magnetic shells effectively reduces interparticle magnetic interactions and thereby enhances their dispersibility and manipulability in solution [83].

As shown in Figure 5 and Table 2, the specific magnetization (*M*_s_) of the surface-functionalized CoFe_2_O_4_ samples at the maximum magnetic field strength (*H* = 10 kOe) exhibits a decrease compared to that of the uncoated CoFe_2_O_4_ nanoparticles during the magnetization process. The reduction in *M*_s_ from 56.9 emu/g for CoFe_2_O_4_ to 51.7 emu/g for F_3_-SiO_2_@CoFe_2_O_4_, 50.3 emu/g for F_9_-SiO_2_@CoFe_2_O_4_, and 28.6 emu/g for F_17_-SiO_2_@CoFe_2_O_4_ can be ascribed to the presence of fluorocarbon chains linked to the homogenous SiO_2_ shell on the surface of the CoFe_2_O_4_ nanoparticles. When the SiO_2_ shell is deposited on the surface of the CoFe_2_O_4_ nanoparticles, a layer is created around the CoFe_2_O_4_ magnetic core that does not contribute to the overall magnetic moment of the nanoparticles. Silicon and oxygen atoms in the SiO_2_ shell lack unpaired electrons, which results in a weaker response of the core–shell nanostructure to an external magnetic field [84,85,86]. On the other hand, fluorocarbon chains that have all their electrons paired behave similarly diamagnetically. The lack of unpaired electrons in these chains means there is no contribution to the net magnetic moment, thus reducing the overall magnetic response of the core–shell structure. The magnetic properties are therefore also affected by the chain length of the fluorocarbons. As seen in Figure 5, longer chains result in a greater reduction in the specific magnetization of the fluoroalkoxysilane-coated CoFe_2_O_4_ nanoparticles than the uncoated CoFe_2_O_4_ nanoparticles.

Further observation revealed a correlation between the increase in both remanent magnetization (*M*_r_) and coercivity (*H*_c_) with increasing particle size. The smallest particles (F_3_-SiO_2_@CoFe_2_O_4_, *D*_TEM_ ≈ 12.7 nm) exhibit the lowest *M*_r_ (8.1 emu/g) and *H*_c_ (152.3 Oe), consistent with a superparamagnetic state. As the particle size increases (F_9_-SiO_2_@CoFe_2_O_4_, *D*_TEM_ ≈ 14.9 nm), both *M*_r_ and *H*_c_ increase (10.1 emu/g and 331.2 Oe, respectively), indicating enhanced magnetic anisotropy associated with stabilized single-domain behavior. This is in line with theoretical expectations, where remanent magnetization (*M*_r_) and coercivity (*H*_c_) increase as long as the particles remain single-domain and their size does not exceed a critical limit (*D* < *D*_(crit)_), typically 10–30 nm for CoFe_2_O_4_ [59,87]. When the particle size drops below ~30 nm (commonly 5–30 nm), superparamagnetic behavior becomes dominant [88]. A slight further decrease in *M*_r_ to 8.1 emu/g and *H*_c_ to 314.1 Oe for the F_17_-SiO_2_@CoFe_2_O_4_ sample (*D*_TEM_ ≈ 18.6 nm; *D*_BET_ ≈ 211.2 nm), as seen in Table 2, is most likely due to increased effective particle size and a lower relative content of magnetically active material, resulting from thicker coatings and stronger aggregation.

Above the critical domain size (*D* > *D_(_*_crit)_), particles tend to form multidomain structures, which lowers the magnetic anisotropy energy and leads to a decrease in coercivity and remanence [87,89,90].

High coercivity is usually characteristic of single-domain particles, as they require a significant magnetic field to reverse the direction of magnetization. In contrast, a low squareness ratio (*M*_r_/*M*_s_), typically below 0.3, indicates soft magnetic or superparamagnetic behavior. In our case, the *M*_r_/*M*_s_ ratio remains < 0.3 for all the prepared nanostructures (Table 2), confirming that more than 70% of the saturation magnetization disappears upon field removal. This behavior is due to thermal fluctuations that randomly change the orientation of magnetization, which is a feature of superparamagnetism. Although the measured coercivity of the uncoated CoFe_2_O_4_ sample is relatively high (*H*_c_ ≈ 269.7 Oe), it remains significantly below the range of 500–2000 Oe reported for strongly anisotropic nanostructures, suggesting that the particles exist near the transition between superparamagnetic and stable single-domain states [91,92]. Thus, the magnetic properties of the prepared nanostructures are the result of a delicate interplay between the size of the magnetic core, the thickness of the non-magnetic shell, and the surface chemistry.

Moreover, the specific magnetization (*M*_s_) also shows a clear size-dependent trend. For the smallest particles (F_3_-SiO_2_@CoFe_2_O_4_), *M*_s_ is 51.7 emu/g, and it remains relatively stable for F_9_-SiO_2_@CoFe_2_O_4_ (50.3 emu/g), but for the largest particles (F_17_-SiO_2_@CoFe_2_O_4_), *M*_s_ drops sharply to 28.6 emu/g. This reduction can be attributed to the presence of a thicker non-magnetic shell and increased particle aggregation, both of which effectively lower the proportion of magnetically active material and thus reduce the overall magnetic response. Such a trend supports the conclusion that the size and functionalization of the surface directly influence the magnetic strength and responsivity of the particles.

This relationship between size and magnetic behavior is crucial for tuning properties for applications such as magnetic separation.

### 2.2. Surface Wettability and Hydrophobicity of Functionalized CoFe_2_O_4_ Nanoparticles

The hydrophobicity or hydrophilicity of the surface is influenced by several factors, including chemical composition, homogeneity, and morphology, depending on the choice of coating materials. Strategies for functionalizing CoFe_2_O_4_ nanoparticles with fluoroalkoxysilanes are based on the number of hydroxyl groups on the surface and their accessibility for binding. The surface becomes hydrophobic when the fluoroalkoxysilanes close all the hydroxyl groups, preventing interactions with water. However, not all hydroxyl groups react with the fluoroalkoxysilanes, leaving free binding sites that allow hydrogen bonds to form. In addition, the lack of anchoring sites for fluoroalkoxysilane groups may result in the CoFe_2_O_4_ core not being fully protected from contact with water [93,94]. Differences in surface composition and functionalization thus affect the wettability of the nanoparticles and determine whether they exhibit hydrophilic or hydrophobic properties.

A simple quantitative method to characterize the relative degree of a liquid interaction with a solid surface is the measurement of a fluid droplet contact angle on a solid substrate [93]. In general, a surface is considered hydrophilic if the contact angle with water is less than 90°, strongly hydrophilic at less than 30°, and superhydrophilic if the contact angle is less than 10°, which causes water to spread out at the surface in a thin layer rather than forming droplets. Conversely, on a hydrophobic surface (contact angle > 90°), water forms distinct droplets, and as hydrophobicity increases, the contact angle also increases. Surfaces with contact angles greater than 150° are classified as superhydrophobic, often exhibiting the Lotus effect, where water droplets easily roll off [94,95]. So, contact angle measurements are a direct indicator of surface wettability and provide insight into the effectiveness of functionalization in altering the hydrophilic or hydrophobic character of the surface.

**Figure 6:**
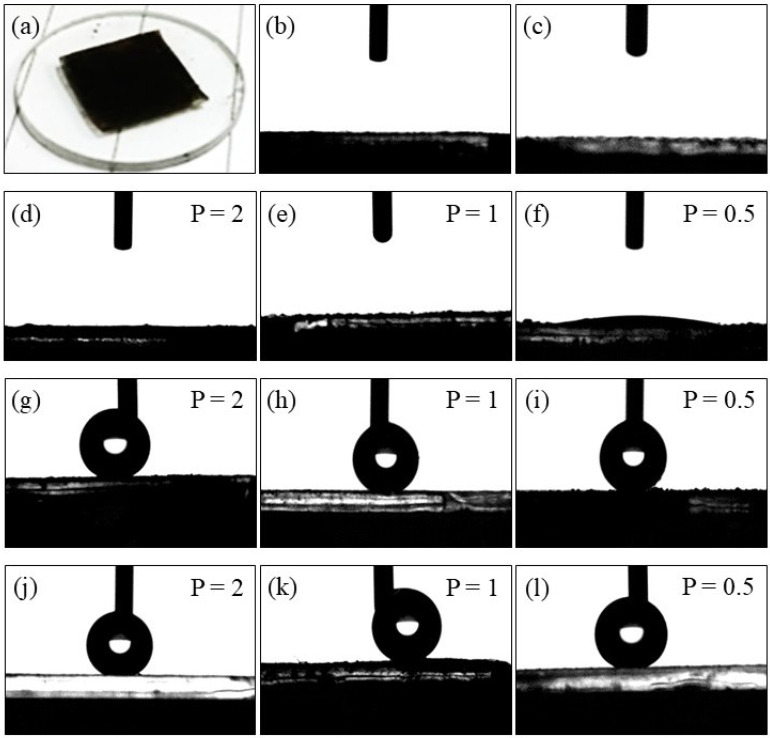
(**a**) Powder compacted onto the surface of a glass slide, and water contact angle measurements for (**b**) CoFe_2_O_4_, (**c**) SiO_2_@CoFe_2_O_4_, (**d**–**f**) F_3_-SiO_2_@CoFe_2_O_4_, (**g**–**i**) F_9_-SiO_2_@CoFe_2_O_4_, and (**j**–**l**) F_17_-SiO_2_@CoFe_2_O_4_ powders at molar ratios of P 2, 1, and 0.5.

Contact angle measurements were performed using the water droplet deposition method, as shown in Figure 6, to assess the wettability and hydrophilic/hydrophobic properties of the synthesized F_3_-SiO_2_@CoFe_2_O_4_, F_9_-SiO_2_@CoFe_2_O_4_, and F_17_-SiO_2_@CoFe_2_O_4_ samples. The exact measurements were also performed on the surfaces of non-functionalized CoFe_2_O_4_ and SiO_2_@CoFe_2_O_4_ nanoparticles. The powders employed for the measurements were compressed onto the glass slide surface (Figure 6a) and subsequently positioned in the contact angle analyzer window. The numerical values of the water contact angle (CA) at the fluoroalkoxysilane-coated CoFe_2_O_4_ surfaces are given in Table 3, while the critical surface tensions (*γ*_c_) were obtained from other sources in the literature [96,97,98] and were not measured in this study.

The findings show that a droplet of deionized water spreads and completely wets the CoFe_2_O_4_ and SiO_2_@CoFe_2_O_4_ surfaces, displaying a 0° contact angle. This behavior is consistent with the strong hydrophilic nature of both surfaces (Figure 6b,c). From an energetic perspective, this means that the adhesion forces involved in the interaction of water with the sample surface surpass the cohesive forces within the bulk state of water [99,100]. This confirms the high wettability of non-functionalized CoFe_2_O_4_ and SiO_2_@CoFe_2_O_4_, which means that their surfaces readily interact with water due to the presence of hydroxyl groups.

As shown in Table 3, the F_3_-SiO_2_@CoFe_2_O_4_ samples also exhibit a highly hydrophilic surface character. These samples have a minimum static water contact angle of 0°, which does not exceed 11.5° for the samples prepared with the highest silane concentration, denoted by P 0.5 (Figure 6d–f). In contrast, samples F_9_-SiO_2_@CoFe_2_O_4_ and F_17_-SiO_2_@CoFe_2_O_4_ exhibit a pronounced hydrophobic behavior, with water forming discrete droplets on their surfaces (Figure 6g–l). For both samples, the contact angle of the water droplets on their surfaces increases considerably with the number of fluorinated carbons, reaching 148.9° for F_9_-SiO_2_@CoFe_2_O_4_ and exceeding 151.3° for F_17_-SiO_2_@CoFe_2_O_4_ (Table 3). This trend of gradually decreasing wettability indicates increasing hydrophobicity associated with longer fluorocarbon chains and a reduced affinity for water.

For the F_3_-SiO_2_@CoFe_2_O_4_ sample, which contains only a few fluorinated carbons, the strong dipole interaction between the water and the surface is caused by the high electronegativity of fluorine and the electropositivity of silicon, which allows for the formation of dipole bonds with the water molecules. In contrast, the F_9_-SiO_2_@CoFe_2_O_4_ and F_17_-SiO_2_@CoFe_2_O_4_ samples with longer fluorinated chains are dominated by sterically hindered, rigid, and non-polar fluorinated segments, which reduce wettability and lead to pronounced hydrophobic or even superhydrophobic behavior.

The inductive effect between the fluorinated groups and the silicon reduces the electron density on the silicon, preventing electron delocalization and limiting the potential for electrostatic interactions with water [93]. The hydrophobicity of these surfaces arises from the fluorinated segments, which cover a large part of the surface and prevent the formation of Van der Waals interactions and hydrogen bonds with water molecules. Consequently, the interactions between the water and the fluorinated surface are predominantly non-electrostatic [94], implying that the hydrophobic behavior is due to the physical characteristics of the surface rather than specific chemical bonds [99].

The polar nature of water enables each water molecule to create up to four hydrogen bonds with other water molecules, an essential trait that typically remains unbroken. Because of cohesive forces, water tends to maintain hydrogen bonds between water molecules rather than interact with hydrophobic surfaces, causing the molecules to stick together and form discrete droplets, thereby reducing contact with the hydrophobic surface. As shown in Figure 6, discrete water droplets assume a spherical shape to minimize surface contact with the fluorinated interfaces in the F_9_-SiO_2_@CoFe_2_O_4_ and F_17_-SiO_2_@CoFe_2_O_4_ samples, reflecting the hydrophobic nature of the fluorinated surfaces. This can be attributed to the fact that the F_9_-SiO_2_@CoFe_2_O_4_ and F_17_-SiO_2_@CoFe_2_O_4_ samples cannot disrupt the hydrogen bonding network characteristic in water. Consequently, there is minimal or no interaction between the water and the strongly fluorinated surfaces of the F_9_-SiO_2_@CoFe_2_O_4_ and F_17_-SiO_2_@CoFe_2_O_4_ samples. This confirms the key role of hydrogen bonds in the interactions between water and fluorinated surfaces, as they directly influence the wettability and adhesion, which, in turn, affect the hydrophobic properties of the material.

### 2.3. Evaluation of Oil Adsorption Capacity and Efficiency Using Functionalized CoFe_2_O_4_ Nanoparticles

**Figure 7:**
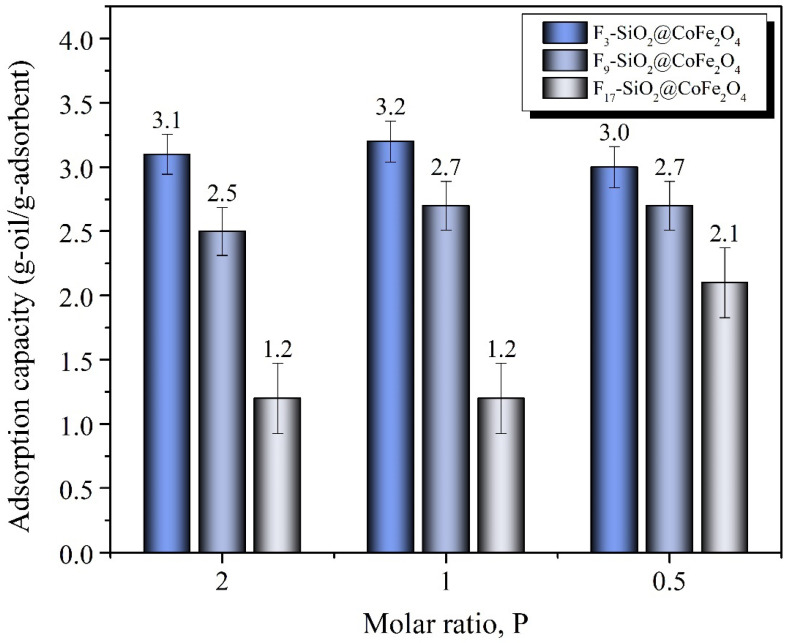
Comparison of the oil adsorption capacity of F_3_-SiO_2_@CoFe_2_O_4_, F_9_-SiO_2_@CoFe_2_O_4_, and F_17_-SiO_2_@CoFe_2_O_4_ nanoparticles at different P molar ratios.

A model of oil-in-water separation was developed to demonstrate the utility of the hydrophobic characteristics of fluorinated CoFe_2_O_4_ nanoparticles. This model aimed to evaluate the performance of fluorinated CoFe_2_O_4_ in removing oil from an oil-in-water mixture. The effectiveness and affinity of the prepared fluorinated magnetic adsorbents F_3_-SiO_2_@CoFe_2_O_4_, F_9_-SiO_2_@CoFe_2_O_4_, and F_17_-SiO_2_@CoFe_2_O_4_ for removing engine oil from the aqueous phase were tested through a series of experiments. Adsorption tests were carried out at pH 4.5 and with a contact time of 5 min. Figure 7 shows the adsorption capacity and efficiency of the individual samples, while Table 4 presents the corresponding numerical data.

Once the fluorinated magnetic adsorbents were prepared, they were added to the oil-in-water mixture to test the oil adsorption capacity and efficiency. When the hydrophobic fluorinated magnetic nanoparticles were attached to the oil droplets and the oil-water interface, the presence of the magnetic nanoparticles caused the oil droplets to become magnetically responsive. The interaction, which promotes the aggregation of the oil droplets, resulted in larger oil aggregates or clusters. The magnetic properties of the nanoparticles were then exploited by introducing an external magnetic field into the system. Oil droplets or aggregates that were magnetically responsive were attracted to the magnetic field and collected in the direction of the magnetic field source. In this way, the magnetic field caused the aggregate oil phase, which was now magnetically responsive due to the presence of magnetic nanoparticles, to move and separate from the aqueous phase.

Oil adsorption results, shown in Table 4, are presented only for functionalized nanoparticles, in particular, F_3_-SiO_2_@CoFe_2_O_4_, F_9_-SiO_2_@CoFe_2_O_4_, and F_17_-SiO_2_@CoFe_2_O_4_, as these nanoparticles typically have a higher adsorption efficiency than their non-functionalized counterparts [101]. As already seen in Figure 6, the non-functionalized CoFe_2_O_4_ and SiO_2_@CoFe_2_O_4_ nanoparticles have a hydrophilic character with low contact angles and a high degree of wettability with water, making them less susceptible to interactions with hydrophobic oil molecules. It has been shown that adding fluorinated hydrocarbon substituents—specifically fluorine or perfluoroalkyl groups—to the hydrophilic surfaces of non-functionalized CoFe_2_O_4_ and SiO_2_@CoFe_2_O_4_ nanoparticles can dramatically change their surface characteristics and thus affect their hydrophilic and oleophilic properties [102].

Depending on the type and structure of the grafted fluorinated chains, these modifications can lead not only to reduced hydrophilicity but even to pronounced superhydrophobic and oleophobic behavior, particularly in the case of long-chain fluoroalkoxysilanes, such as in the F_17_-SiO_2_@CoFe_2_O_4_ sample.

The mechanism behind this observed superhydrophobicity and oleophobicity can be explained by a synergistic interplay between two main factors: (i) surface chemical composition and (ii) surface morphology at the micro- and nanoscale.

The increasing length of fluorinated alkyl chains (e.g., F_17_-SiO_2_@CoFe_2_O_4_) reduces the surface energy due to the high density of terminal –CF_2_– and –CF_3_ groups, which are both low-energy and highly non-polar. These fluorinated moieties reduce interaction with water and non-polar liquids, resulting in higher contact angles and improved repellency. Furthermore, longer chains promote denser, uniform surface coverage, minimizing wetting pathways and enhancing the liquid-repellent barrier.

Surface morphology also plays a crucial role. As can be seen from the TEM analyses (Figure 2), the functionalized nanoparticles have hierarchical surface structures consisting of a CoFe_2_O_4_ magnetic core coated with a SiO_2_ shell and an outer layer of fluorinated silane groups, which are capable of trapping air below the liquid droplet.

This condition is well described by the Cassie–Baxter wetting regime [103], in which the droplet rests on a composite interface of solid and air, leading to high apparent contact angles and low adhesion. If the liquid fully penetrates the surface texture, wetting is better described by Wenzel’s model [104], where increased roughness increases the effective solid–liquid contact area and thus enhances intrinsic wetting, which promotes spreading on hydrophilic surfaces or increases repulsion on hydrophobic ones. Although this study did not explicitly model based on the Cassie–Baxter or Wenzel equations, the observed high contact angle values and hierarchical surface morphology suggest that Cassie–Baxter-type wetting is likely dominant in our system. However, this interpretation is still qualitative and should be considered speculative, based solely on circumstantial evidence from microscopy and wettability data. Local transitions to the Wenzel regime cannot be excluded, especially in less uniformly functionalized or more densely textured areas. The combined effects of fluorinated surface chemistry and hierarchical surface roughness lead to the pronounced superhydrophobic and oleophobic properties observed for F_17_-SiO_2_@CoFe_2_O_4_. However, as the data in Table 4 indicate, these characteristics are associated with lower oil adsorption capacities compared to shorter-chain analogues (F_3_- and F_9_-SiO_2_@CoFe_2_O_4_), illustrating an inverse relationship between chain-length-induced repellency and oil affinity.

The systematic oil adsorption tests underline the remarkable adsorption capacity of the F_3_-SiO_2_@CoFe_2_O_4_ sample, which can adsorb an average of 3.1 g oil/g adsorbent and achieve an average adsorption efficiency of 98%. This is an excellent achievement compared to other documented studies [105,106,107]. It can be observed from Table 4 that the adsorption capacity of the F_3_-SiO_2_@CoFe_2_O_4_ sample remains almost unchanged, ranging from 3.0 to 3.2 g oil/g adsorbent, even though the concentration of the alkoxysilane FTPTMS in the sample increases when the molar ratio P changes from 2 to 0.5. This indicates that the surface area of the F_3_-SiO_2_@CoFe_2_O_4_ nanoparticles may have reached the maximum possible density of bound FTPTMS molecules. When the surface of the nanoparticles is saturated, the additional FTPTMS molecules do not contribute to increasing the number of accessible fluorine sites for adsorption, which keeps the adsorption capacity unchanged. In addition, high concentrations of FTPTMS cause steric hindrance and increased molecular density on the surface, which may limit the access of oil molecules to the active sites. At even higher concentrations, nanoparticle aggregation may occur, further reducing the adsorption efficiency due to the reduced accessibility of the functional surfaces. This could explain the slight decrease in the average adsorption capacity of the F_3_-SiO_2_@CoFe_2_O_4_ sample from 3.2 to 3.0 g oil/g adsorbent when the concentration of FTPTMS is increased by changing the molar ratio P from 1 to 0.5. Comparison of the remaining adsorption results in Table 4 reveals that the nanoparticle samples F_9_-SiO_2_@CoFe_2_O_4_ and F_17_-SiO_2_@CoFe_2_O_4_ exhibit a much lower oil adsorption capacity compared to the F_3_-SiO_2_@CoFe_2_O_4_ sample in the order of *Q*_ads_(F_17_-SiO_2_@CoFe_2_O_4_) < *Q*_ads_(F_9_-SiO_2_@CoFe_2_O_4_) < *Q*_ads_(F_3_-SiO_2_@CoFe_2_O_4_) for a given molar value of P. The higher oleophobicity of the F_9_-SiO_2_@CoFe_2_O_4_ and F_17_-SiO_2_@CoFe_2_O_4_ samples is most likely due to the difference in the length of the fluorocarbon chains and the number of fluorine atoms in the fluorocarbon chain structure of the fluoroalkoxysilanes NFHTMS and PFDTES.

It can also be seen from the data in Table 4 that the adsorption capacity of both samples, F_9_-SiO_2_@CoFe_2_O_4_ and F_17_-SiO_2_@CoFe_2_O_4_, increases with increasing concentrations of NFHTMS and PFDTES fluoroalkoxysilanes when the molar ratio of P decreases from 2 to 0.5. For sample F_9_-SiO_2_@CoFe_2_O_4_, the adsorption capacity increases slightly from 2.5 to 2.8 g oil/g adsorbent. In contrast, sample F_17_-SiO_2_@CoFe_2_O_4_, despite following a similar trend, exhibits a noticeably lower adsorption capacity, ranging from 1.2 g oil/g adsorbent at P 2 to 2.1 g oil/g adsorbent at P 0.5.

In these cases, the trend clearly shows a positive correlation between the adsorption capacity and the concentration of fluoroalkoxysilanes in each sample. While an increase in the concentration of NFHTMS and PFDTES fluoroalkoxysilanes in the F_9_-SiO_2_@CoFe_2_O_4_ and F_17_-SiO_2_@CoFe_2_O_4_ samples leads to an increase in the adsorption capacity, experimental data suggest that the length of the fluorocarbon chain and the number of fluorine atoms in the fluoroalkoxysilane structure also have an important influence on the adsorption dynamics.

The results in Table 4 confirm that the higher number of fluorine atoms in fluoroalkoxysilanes contributes to the higher hydrophobicity and oleophobicity of the material, which is reflected in the lower oil adsorption observed in the F_9_-SiO_2_@CoFe_2_O_4_ and F_17_-SiO_2_@CoFe_2_O_4_ samples compared to F_3_-SiO_2_@CoFe_2_O_4_. These observations are consistent with the physicochemical properties of fluorine: its high electronegativity (*χ* = 3.98 by Pauling), high ionization energy (17.42 eV), and low polarizability (*α*_D_ = 3.74) strongly influence the intermolecular interactions of fluorocarbons and consequently reduce the adsorption of oil molecules [108,109]. It is known that weak London dispersion forces govern the interactions between fluorocarbons and hydrocarbons in water, the effectiveness of which is strongly dependent on the polarizability of the molecules [110]. Since the C-F bond is extremely stable and highly polarized, the fluorocarbon chains have a low affinity for dispersion interactions with oils, which directly reduces the adsorption of oil molecules onto the surfaces of fluorinated nanoparticles [111]. This trend is even more evident when comparing the F_9_-SiO_2_@CoFe_2_O_4_ and F_17_-SiO_2_@CoFe_2_O_4_ samples with the F_3_-SiO_2_@CoFe_2_O_4_ sample. Adsorbents with longer fluorocarbon chains (F_9_-SiO_2_@CoFe_2_O_4_ and F_17_-SiO_2_@CoFe_2_O_4_) and a higher degree of fluorination show a significantly lower adsorption capacity. This reduction can be attributed to the increased intramolecular cohesion and decreased polarizability of the longer fluorinated chains, which further limits the efficiency of dispersion interactions with the oil molecules and reduces their ability to bind to the functionalized surface.

In addition, hydrophobic solvation can also influence oil adsorption in aqueous environments [112]. In the presence of fluorocarbon chains, water molecules reorganize and form stable solvation structures (“water cages”) that isolate the fluorocarbon chains and limit their interaction with the oil. This effect is more pronounced in the F_9_-SiO_2_@CoFe_2_O_4_ and F_17_-SiO_2_@CoFe_2_O_4_ samples, where the longer fluorocarbon chains promote the formation of more stable hydration structures, further reducing their oil adsorption capacity [113,114,115]. These results confirm that oil adsorption on fluoroalkoxysilane functionalized surfaces is strongly dependent on the length and electronic properties of the fluorocarbon chains. Longer and more fluorinated chains reduce adsorption due to a combination of lower polarizability, stronger intermolecular cohesion, and hydrophobic solvation, which is an important factor in the design of oleophobic materials.

In addition to the effects associated with hydrophobic solvation, another key property of fluorocarbons is their extremely low critical surface tension (*γ*_c_), which results from strong intramolecular cohesiveness and weak intermolecular forces. This low surface tension further limits the interactions between the fluorocarbon chains and the oil molecules, which is confirmed by the trend of reduced oil adsorption capacity in the F_9_-SiO_2_@CoFe_2_O_4_ and F_17_-SiO_2_@CoFe_2_O_4_ samples. This property is key to understanding the oleophobic behavior of fluorocarbons, as it implies their resistance to oil wetting [96,97,116]. Fluorocarbons have very low surface tension due to the high electronegativity of the fluorine atoms in their structure. The propensity of oil to wet the surface of fluorocarbons, therefore, depends on the difference between the surface tension of the oil and the critical surface tension of the fluorocarbons. If the surface tension of the oil is less than or approximately equal to the critical tension of the fluorocarbons, the oil will expand and wet the surface more readily. However, if the surface tension of the oil is much higher, the oil remains in the form of droplets and does not effectively wet the surface.

As part of the study, the surface tension of waste Quartz INEO MC3 5W-30 engine oil in water was measured using the stalagmometric method, yielding a value of 31.9 ± 0.4 mN/m. The oil density was determined to be 0.829 ± 0.012 g/cm^3^. Based on these measured data and taking into account the available literature data on the critical surface tensions of fluoroalkoxysilanes, which are 33.5 mN/m for TFPTMS, 23 mN/m for NFHTMS, and 12 mN/m for PFDTES [96,98,116], it can be concluded that engine oil will wet and disperse more efficiently over the surface of the F_3_-SiO_2_@CoFe_2_O_4_ sample. This is due to the favorable ratio of the oil surface tension to the critical surface tension of the solid phase, which allows for greater intermolecular interactions and consequently better adsorption of oil molecules. In contrast, a more pronounced oleophobic character is expected for the samples F_9_-SiO_2_@CoFe_2_O_4_ and F_17_-SiO_2_@CoFe_2_O_4_ functionalized with NFHTMS and PFDTES, respectively, which have a lower critical surface tension. This results in less efficient interactions between the oil and the surface, which is reflected in a reduced adsorption of oil molecules. The experimental results in Table 4 support these hypotheses, since the adsorption of oil molecules on the samples studied systematically decreases in the order F_3_-SiO_2_@CoFe_2_O_4_ > F_9_-SiO_2_@CoFe_2_O_4_ > F_17_-SiO_2_@CoFe_2_O_4_. This means that the F_3_-SiO_2_@CoFe_2_O_4_ sample shows the lowest oleophobicity, allowing for a better wetting effect and higher oil adsorption. The F_9_-SiO_2_@CoFe_2_O_4_ sample shows intermediate properties, having a slightly higher oleophobicity compared to F_3_-SiO_2_@CoFe_2_O_4_, leading to reduced wetting and consequently lower oil adsorption. On the other hand, the F_17_-SiO_2_@CoFe_2_O_4_ sample shows a markedly limited ability to bind oil molecules due to the highest oleophobicity. Figure 7 visually shows the adsorption capacity and efficiency of the samples.

### 2.4. Oil Desorption and Regeneration of Adsorbents

Adsorbent regeneration and reusability after adsorption are essential steps that have positive environmental effects because they reduce waste production by taking care of spent adsorbent disposal issues and lower the need for new adsorbents [117,118,119].

The present study performed regeneration experiments for three selected functionalized nanocomposites, specifically F_3_-SiO_2_@CoFe_2_O_4_, F_9_-SiO_2_@CoFe_2_O_4_, and F_17_-SiO_2_@CoFe_2_O_4_. In the regeneration process, ethanol was used as a solvent to extract oil from the adsorbent surface, as is commonly applied in similar studies [105,106]. Various oil desorption strategies exist, including biological, thermal, chemical, and photochemical processes [120]; however, solvent-based regeneration using ethanol was selected for its simplicity, efficiency, and compatibility with the surface chemistry of the materials.

In this investigation, reusability was evaluated over three successive oil adsorption–desorption cycles, corresponding to the established methodology for the initial stability assessment of new adsorbents, as reported in other studies [121,122].

Although desorption performance can also be monitored continuously over time using flow-through systems, our experiments were conducted in batch mode, which provides better control of adsorption and regeneration conditions and allows for straightforward comparison between different materials. Batch systems are particularly suitable for the early-phase evaluation of adsorbent reusability and are commonly employed for such purposes [121,122,123].

**Figure 8:**
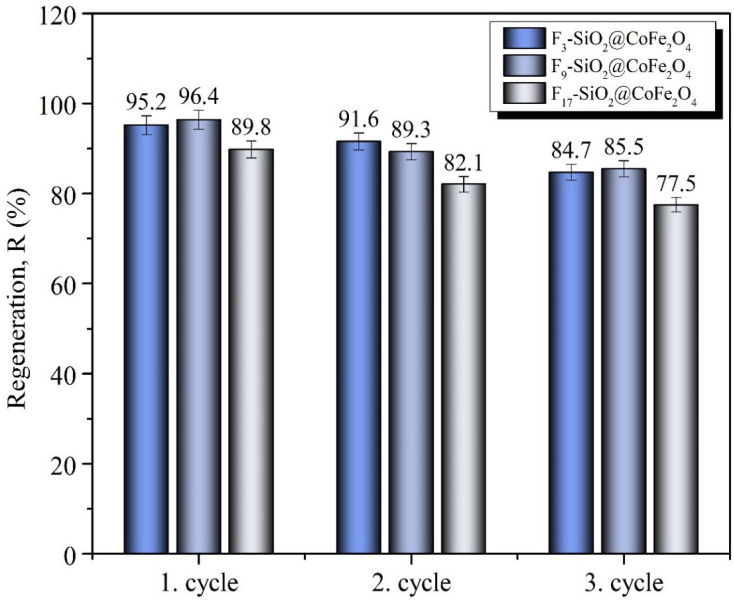
The average oil desorption efficiency of F_3_-SiO_2_@CoFe_2_O_4,_ F_9_-SiO_2_@CoFe_2_O_4_, and F_17_-SiO_2_@CoFe_2_O_4_ adsorbents over three successive adsorption–desorption cycles.

The regeneration experiments were performed under consistent conditions established at 50 mL of 0.5 vol.% oil–water mixture and 50 mg of the adsorbent per cycle. Figure 8 illustrates the regeneration performance of the F_3_-SiO_2_@CoFe_2_O_4_, F_9_-SiO_2_@CoFe_2_O_4_, and F_17_-SiO_2_@CoFe_2_O_4_ samples over three consecutive adsorption–desorption cycles, with the corresponding oil removal efficiencies (*R*_e_ [%]) summarized in Table 5.

The results show a gradual decrease in oil removal efficiency between cycles, mainly due to surface saturation, reduced availability of active sites, and potential changes in surface wettability due to repeated exposure to ethanol. For F_3_-SiO_2_@CoFe_2_O_4_, the removal efficiency decreased from 95.2% in the first cycle to 91.6% in the second and 84.7% in the third. Similarly, for F_9_-SiO_2_@CoFe_2_O_4_, the efficiency decreased from 96.4% in the first cycle to 89.3% in the second and 85.5% in the third cycle, while for F_17_-SiO_2_@CoFe_2_O_4_, the efficiency decreased from 89.8% to 82.1% and then to 77.5%, as shown in Figure 8 and Table 5. In each adsorption–desorption cycle, desorption was carried out in batch mode by immersing the oil-loaded adsorbents in ethanol for a fixed duration of 5 min. This timed step allowed for oil extraction from the surface of F_3_-SiO_2_@CoFe_2_O_4_, F_9_-SiO_2_@CoFe_2_O_4_, and F_17_-SiO_2_@CoFe_2_O_4_. Despite the observed decrease in desorption efficiency, all adsorbents retained high functionality and structural stability after three cycles, confirming their potential for reuse and practical use in oil–water separation.

## 3. Materials and Methods

**Figure 9:**
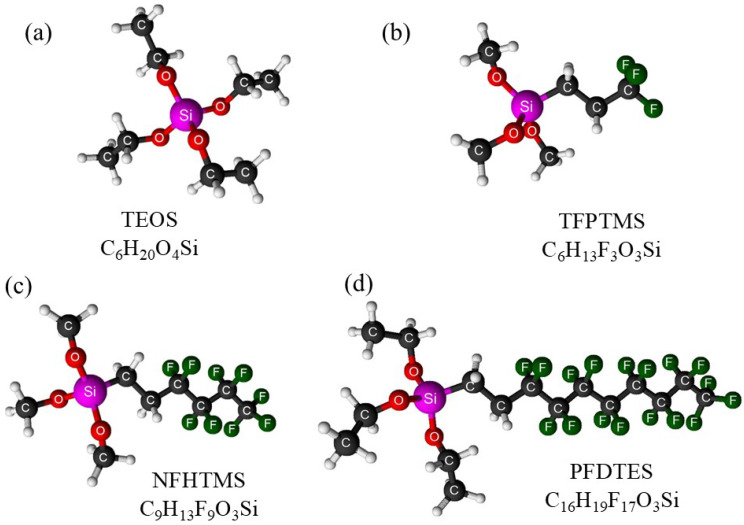
Three-dimensional molecular structures of the investigated alkoxysilanes: (**a**) TEOS, (**b**) TFPTMS, (**c**) NFHTMS, and (**d**) PFDTES.

All the chemicals used in this study were generally of reagent grade and obtained from commercial sources without further purification: cobalt (II) chloride hexahydrate (CoCl_2_⋅6H_2_O, 98%, 237.93 g/mol, CAS No. 7791-13-1, SigmaAldrich, St. Louis, MO, USA), iron (III) chloride hexahydrate (FeCl_3_⋅6H_2_O, ≥98%, 270.3 g/mol, CAS No. 10025-77-1, SigmaAldrich), sodium hydroxide (NaOH, ≥98% (anhydrous), 40 g/mol, CAS No. 1310-73-2, SigmaAldrich), 2-propanol (C_3_H_8_O, 99.8%, 60.1 g/mol, 0.785 g/mL, CAS No. 67-63-0, GramMol, Zagreb, Croatia), ethanol (C_2_H_5_OH, 96%, 46.07 g/mol, 0.810 g/mL, CAS No. 64-17-5, GramMol), tetraethyl orthosilicate, TEOS (C_6_H_20_O_4_Si, 99%, 208.33 g/mol, 0.94 g/mL, CAS No. 78-10-4, SigmaAldrich), trimethoxy(3,3,3-trifluoropropyl)silane, TFPTMS (C_6_H_13_F_3_O_3_Si, ≥97.0%, 218.25 g/mol, 1.142 g/mL, CAS No. 429-60-7, SigmaAldrich), trimethoxy(1H,1H,2H,2H-nonafluorohexyl)silane, NFHTMS (C_9_H_13_F_9_O_3_Si, 98%, 368.27 g/mol, 1.35 g/mL, CAS No. 85877-79-8, Tokyo Chemical Industry Co., Ltd., Tokyo, Japan), and triethoxy(1H,1H,2H,2H-perfluorodecyl)silane, PFDTES (C_16_H_19_F_17_O_3_Si, 97%, 610.38 g/mol, 1.389 g/mL, CAS no. 101947-16-4, SigmaAldrich). Figure 9 illustrates the 3D structures of the alkoxysilanes used in this study. A waste commercially available low-SAPS (Sulfated Ash, Phosphorus, and Sulfur) synthetic engine oil, Quartz INEO MC3 5W-30 (TotalEnergies, Inc., Courbevoie, France), was also used. The physicochemical properties of Quartz INEO MC3 5W-30 are detailed in Ref. [124]. Deionized water (dH_2_O) was used to prepare all suspensions and solutions.

### 3.1. Synthesis of Magnetic Nanoparticles

#### 3.1.1. Magnetic CoFe_2_O_4_ Nanoparticles

Cobalt ferrite (CoFe_2_O_4_) nanoparticles were obtained by the coprecipitation method using Co^2+^ and Fe^3+^ salts in an alkaline aqueous medium at a moderately elevated temperature, following Schikorr’s reaction [125], as shown in Equation (4):

Co^2+^ + 2Fe^3+^ + 4OH^−^ + O_2_ = CoFe_2_O_4(s)_ + 2H_2_O (4)

The synthesis of CoFe_2_O_4_ magnetic nanoparticles involved the preparation of stock aqueous solutions containing Co^2+^ and Fe^3+^ ions using CoCl_2_⋅6H_2_O and FeCl_3_⋅6H_2_O as precursor reagents. Stoichiometric amounts of the appropriate chlorides were dissolved in deionized water to obtain a solution with a concentration of 0.5 M. This solution was then added to 0.5 M aqueous sodium hydroxide solution preheated to (87 ± 2) °C under reflux and stirred at 400 rpm. The reaction proceeded at pH 10 for at least one hour. After completion, the dark brown CoFe_2_O_4_ precipitate was thoroughly washed with deionized water and separated from the supernatant by magnetic decantation. This washing procedure was repeated several times to ensure that all the remaining reactants were removed. After washing, the precipitate was dried in a laboratory oven at 90 °C for 24 h. This precise procedure ensured the production of high-quality CoFe_2_O_4_ nanoparticles.

#### 3.1.2. Magnetic Fluoroalkoxysilane-Coated CoFe_2_O_4_ Nanoparticles

For the functionalization of CoFe_2_O_4_ nanoparticles with fluoroalkoxysilanes (TFPTMS, NFHTMS, and PFDTES), an in situ method was used, in which the reactants were combined in the following molar proportions: 43.2% 2-propanol, 56.1% distilled water, 0.548% of a 25% NH_4_OH solution, 6 mL of a pre-prepared aqueous colloidal suspension of CoFe_2_O_4_ with a mass concentration (*γ*_i_) of 0.0914 ± 0.005 g/mL, and 0.056% TEOS. The fluoroalkoxysilanes were separately incorporated into the reaction mixture following a molar ratio of TEOS to fluoroalkoxysilanes (P) of 1:2, 1:1, and 2:1, which are hereafter referred to as P 0.5, 1, and 2, respectively.

In the presence of a base (NH_4_OH), hydrolysis and condensation of the alkoxysilanes occurred, forming siloxane bonds that formed a protective layer around the nanoparticles. The general chemical reaction describing this process is as follows (Equation (5)):

(5) $$m\left({\left(R”O\right)}_{3}Si{\left(CH_{2}\right)}_{2}-R\right)+n\left(Si{\left(OR”\right)}_{4}\right)\overset{+H_{2}O/-R”OH}{\to }\left[{\left(SiO_{2}\right)}_{n}{\left(SiO_{1.5}{\left(CH_{2}\right)}_{2}-R\right)}_{m}\right]$$

where *R* = –(CF_2_)_x_CF_3_ (x = 0, 3, 7) and *R”* = –CH_3_ or –CH_2_CH_3_.

The reaction mixture was strongly stirred magnetically at ambient temperature and 500 rpm for 24 h in a closed glass container. After the reaction, the precipitate was thoroughly washed several times with ethanol (96 wt.%) and distilled water.

The resulting magnetic core–shell nanoparticles, designated as F_3_-SiO_2_@CoFe_2_O_4_, F_9_-SiO_2_@CoFe_2_O_4_, and F_17_-SiO_2_@CoFe_2_O_4_, were isolated from the supernatant with an external permanent magnet and then dried overnight in a laboratory oven at 90 °C.

### 3.2. Characterization of Nanoparticles

The prepared samples underwent various characterization techniques, including X-ray diffractometry (XRD), transmission electron microscopy (TEM) paired with energy-dispersive X-ray spectroscopy (EDXS), the Brunauer–Emmet–Teller specific surface area technique (BET), Fourier transform infrared spectroscopy (FTIR), contact angle (CA) measurements, and vibrating sample magnetometry (VSM).

#### 3.2.1. X-Ray Diffractometry (XRD)

X-ray diffractometry (XRD) was performed for structural analysis using a Brucker D4 Endeavor X-ray diffractometer (Billerica, MA, USA) combined with CuKα-radiation. Measurements were made at room temperature in 30 s, ranging from a Bragg angle of 2*θ* at 20° to 80° with an angular step of 0.036°. XRD was performed using a Cu anode with a wavelength of 0.154 nm.

#### 3.2.2. Transmission Electron Microscopy (TEM) with Energy Dispersive X-Ray Spectroscopy (EDXS)

TEM images were taken with a JEOL JEM-2100 microscope (Akishima, Japan). The nanoparticle suspension was dropped onto a thin carbon-coated copper grid and dried at room temperature. TEM analysis combined with EDXS was performed with an accelerating voltage of 200 keV.

#### 3.2.3. Fourier Transform Infrared Spectroscopy (FT-IR)

FT-IR data were collected using Spectrum Two FT-IR spectrometer (PerkinElmer, Waltham, MA, USA) utilizing a KBr window to collect data in the spectral range from 400 cm^−1^ to 4000 cm^−1^ at a resolution of 0.5 cm^−1^. FT-IR spectra were recorded with the PerkinElmer Spectrum 10™ software (Application version 10.5.4.738, released in 2016) at room temperature in transmittance mode.

#### 3.2.4. Brunauer, Emmet, and Teller Method (BET)

BET was used to determine the specific surface area of the nanoparticles using Micromeritics, Flow Prep 060 with Tristar II 3020 (Micromeritics Instrument Corporation, Norcross, GA, USA). All samples were degassed at 110 °C for 24 h before each measurement. The specific surface area was measured in the 0.05–0.3 range of relative pressure in nitrogen gas at a temperature of 77.35 K after 24 h.

#### 3.2.5. Vibrating Sample Magnetometry (VSM)

A Lake Shore 7400 vibrating sample magnetometer (Lake Shore Cryotronics, Inc, Westerville, OH, USA) was used to measure mass magnetization *M* (emu/g) as a function of the applied magnetic field *H* (Oe) for all prepared samples at room temperature. The measurements were carried out in the magnetic field strength range from −10 kOe to 10 kOe.

#### 3.2.6. Contact Angle (CA) Measurements

Static contact angle measurements were performed by uniformly applying a sample of adsorbent nanoparticles to a flat glass substrate and using a metal tube on a goniometer to apply a drop of water of a specific volume to the surface. By analyzing the image, we monitored the change in the height, surface, volume, and diameter of the water drop and calculated the contact angle of the water drop with the surface using Equation (6):

(6) $$\tan\frac{\theta }{2}=\frac{2\cdot h_{k}}{d_{k}}$$

where *h*_k_ (mm) is the height of the water droplet, *d*_k_ (mm) is the width of the water droplet, and *θ* (º) is the contact angle.

#### 3.2.7. Surface Tension Measurements

The surface tension of the waste engine oil was determined using the stalagmometric method, with water as the reference liquid. The number of droplets formed from a given volume of each liquid was recorded, and the surface tension was calculated using Equation (7):

(7) $$\frac{\gamma_{oil}}{\gamma_{water}}=\frac{n_{water}\cdot \rho_{oil}}{n_{oil}\cdot \rho_{water}}$$

where *γ* (mN/m) is the surface tension, *n* is the number of droplets, and *ρ* (g/cm^3^) is the density of the liquid. Each measurement was performed five times to ensure the accuracy and reproducibility of the results. The experiment was performed at room temperature under identical conditions for all samples to ensure the reliability of the measurements.

### 3.3. Oil Adsorption and Desorption Tests

The adsorption study was performed according to the analytical procedure established in our laboratory. Roughly 0.25 mL of waste engine oil was dispersed into 50 mL of water in individual glass containers. Subsequently, 50 mg of each synthesized adsorbent nanoparticle was introduced, namely F_3_-SiO_2_@CoFe_2_O_4_, F_9_-SiO_2_@CoFe_2_O_4_, and F_17_-SiO_2_@CoFe_2_O_4_. Adsorption was performed in triplicate for 5 min at room temperature. A permanent magnet was then placed near the water surface to attract and remove the magnetic adsorbent nanoparticles with the adsorbed oil, and adsorption capacity was determined gravimetrically by the following Equation (8):

(8) $$Q_{ads}=\frac{\left(m_{2}-m_{3}\right)}{m_{1}}$$

where *m*_1_ is the mass of the adsorbent (g), *m*_2_ is the total mass of the beaker containing the water and the oil (mg), *m*_3_ is the total mass of the beaker containing water and oil residue (mg), and *Q*_ads_ is the adsorption capacity (mg/g).

Furthermore, the desorption of oil adsorbed at the surface of the adsorbent nanoparticles was performed by mixing adsorbent nanoparticles with ethanol (96 wt.%) at 25 °C for 5 min in an ultrasound bath. Adsorbent nanoparticles were afterward settled out and separated from the effluent (ethanol-containing desorbed oil) using an external permanent magnet. The adsorbent nanoparticles were thoroughly washed with ethanol and dried for 2 h in a laboratory oven at 120 °C. The desorption efficiency, *R*_e_ (%), was determined gravimetrically using Equation (9):

(9) $$R_{e}(\%)=\frac{C_{des}}{C_{ads}}\cdot 100$$

where *C*_des_ (mg/g) is the concentration of adsorbate desorbed, and *C*_ads_ (mg/g) is the concentration of adsorbate adsorbed.

## 4. Conclusions

In the present study, we developed and evaluated efficient (super)hydrophobic adsorbents based on magnetic cobalt ferrite nanoparticles, CoFe_2_O_4_, functionalized with three different fluoroalkoxysilanes: trimethoxy(3,3,3-trifluoropropyl)silane (TFPTMS), trimethoxy(1H,1H,2H,2H-nonafluorohexyl)silane (NFHTMS), and triethoxy(1H,1H,2H,2H-perfluorodecyl)silane (PFDTES). These fluorosilanes differ in the length of the carbon chains and in the number and arrangement of fluorinated groups (–CF_2_–, –CF_3_), significantly affecting the surface energy and thus the adsorption properties of the prepared materials.

The study aimed to investigate in detail how these structural differences affect the hydrophilic–hydrophobic properties of the prepared materials and to evaluate their ability to adsorb waste engine oil from the aqueous phase. The prepared spherical magnetic nanoparticles F_3_-SiO_2_@CoFe_2_O_4_, F_9_-SiO_2_@CoFe_2_O_4_, and F_17_-SiO_2_@CoFe_2_O_4_ exhibited favorable structural, morphological, and magnetic properties, as confirmed by XRD, TEM/EDXS, FTIR, BET, and contact angle measurements.

The results showed that the adsorption efficiency of the prepared materials was inversely proportional to the length of the fluorocarbon chains. The sample F_3_-SiO_2_@CoFe_2_O_4_, functionalized with the short-chain precursor TFPTMS, achieved the highest adsorption capacity (3.1 g oil/g adsorbent), followed by F_9_-SiO_2_@CoFe_2_O_4_ (2.7 g oil/g adsorbent) and F_17_-SiO_2_@CoFe_2_O_4_ (1.5 g oil/g adsorbent). The short fluorocarbon chains of TFPTMS resulted in reduced hydrophobicity and increased oleophilicity of the F_3_-SiO_2_@CoFe_2_O_4_ sample, as confirmed by contact angles of less than 10°. In contrast, the longer chains of the NFHTMS and PFDTES precursors resulted in a pronounced (super)hydrophobic and oleophobic character of the F_9_-SiO_2_@CoFe_2_O_4_ and F_17_-SiO_2_@CoFe_2_O_4_ samples, with contact angles exceeding 148° and 151°, respectively.

One of the key advantages of the prepared materials is their magnetic responsiveness, which allows for fast and easy separation from the aqueous phase using an external magnetic field. The experimental data also showed a high oil desorption efficiency (77–97%), confirming the possibility of repeated use in successive adsorption/desorption cycles. However, the efficiency of such systems may depend on various factors, such as the size and stability of the nanoparticles, the physicochemical properties of the fluorocarbon coating, and the composition of the oil–water mixtures. Therefore, for further improvements in the performance and selectivity of these adsorbents, it is crucial to understand the mechanisms of interactions between fluorinated magnetic materials and oil mixtures in water to thoroughly understand their performance. However, the development of such materials and technologies should not ignore environmental aspects, particularly the persistence of fluorinated compounds, their potential for bioaccumulation, and possible adverse effects on health and the environment. Overall, the findings of this study not only contribute to the understanding of the influence of specific chemical modifications on the adsorption behavior of materials but also provide a starting point for the development of selective adsorbents and advanced surface coatings.

## Figures and Tables

**Table 1:** Specific surface area (*S*_A_) and the size (*D*_BET_) of the prepared fluoroalkoxysilane-coated CoFe_2_O_4_ nanoparticles.

Molar Ratio (*P*)	Fluoroalkylsilane-Coated CoFe_2_O_4_ Nanoparticles					
	F_3_-SiO_2_@CoFe_2_O_4_		F_9_-SiO_2_@CoFe_2_O_4_		F_17_-SiO_2_@CoFe_2_O_4_	
	*S*_A_ (m^2^/g)	*D*_BET_ (nm)	*S*_A_ (m^2^/g)	*D*_BET_ (nm)	*S*_A_ (m^2^/g)	*D*_BET_ (nm)
2.0	94.4 ± 0.5	11.86 ± 0.06	92.1 ± 0.5	12.15 ± 0.07	44.9 ± 0.2	24.93 ± 0.11
1.0	100.0 ± 0.4	11.19 ± 0.04	77.1 ± 0.4	14.52 ± 0.08	5.3 ± 0.1	211.20 ± 4.06
0.5	101.3 ± 0.4	11.05 ± 0.04	70.8 ± 0.3	15.81 ± 0.07	0.8 ± 0.1	1399.30 ± 199.90

**Table 2:** Comparative analysis of magnetic properties for CoFe_2_O_4_ nanoparticles functionalized with TFPTMS, PFDTES, and NFHTMS alkoxysilanes at a molar ratio of P 1.

Sample	Specific Magnetization (*M*_s_) [emu/g]	Retentivity (*M*_r_) [emu/g]	Coercivity (*H*_c_) [Oe]	Squareness Ratio (*M*_r_/*M*_s_)
CoFe_2_O_4_	56.9	15.2	269.7	0.27
SiO_2_@CoFe_2_O_4_	52.9	15.0	318.3	0.28
F_3_-SiO_2_@CoFe_2_O_4_	51.7	8.1	152.3	0.16
F_9_-SiO_2_@CoFe_2_O_4_	50.3	10.1	331.2	0.20
F_17_-SiO_2_@CoFe_2_O_4_	28.6	8.1	314.1	0.28

**Table 3:** Water contact angle (CA) and critical surface tension (*γ*_c_) at the fluoroalkoxysilane-coated CoFe_2_O_4_ surfaces.

Adsorbent Nanoparticles	Contact Angle, CA (Degree)			Critical Surface Tension *γ*_c_ (mN/m) *
	Molar Ratio (*P*)			
	2	1	0.5	
F_3_-SiO_2_@CoFe_2_O_4_	0	0	11.5	33.5
F_9_-SiO_2_@CoFe_2_O_4_	148.9	149.9	152.7	23
F_17_-SiO_2_@CoFe_2_O_4_	151.3	153.1	159.2	12

* From the authors’ work, Brzoska et al. [96], Yoshino et al. [97], and Zisman et al. [98].

**Table 4:** An average oil adsorption capacity, *Q*_ads_ (g-oil/g-adsorbent), of the prepared adsorbents.

Adsorbent Nanoparticles	Average Oil Adsorption Capacity, *Q_ads_* [g/g]		
	Molar Ratio (*P*)		
	2	1	0.5
F_3_-SiO_2_@CoFe_2_O_4_	3.12 ± 0.15	3.21 ± 0.16	3.01 ± 0.16
F_9_-SiO_2_@CoFe_2_O_4_	2.51 ± 0.18	2.74 ± 0.19	2.78 ± 0.19
F_17_-SiO_2_@CoFe_2_O_4_	1.22 ± 0.27	1.25 ± 0.27	2.11 ± 0.27

**Table 5:** The average oil desorption efficiency, *R*_e_ (%), with standard deviations (*n* = 3) over three successive adsorption–desorption cycles.

Adsorbent Nanoparticles	Average Oil Desorption Efficiency, *R*_e_ (%), with Standard Deviations (*n* = 3)		
	1st Cycle	2nd Cycle	3rd Cycle
F_3_-SiO_2_@CoFe_2_O_4_	95.2 ± 2.1	91.6 ± 1.9	84.7 ± 1.8
F_9_-SiO_2_@CoFe_2_O_4_	96.4 ± 2.1	89.3 ± 1.8	85.5 ± 1.8
F_17_-SiO_2_@CoFe_2_O_4_	89.9 ± 1.9	82.1 ± 1.7	77.5 ± 1.6

## Data Availability

The raw data supporting the conclusions of this article will be made available by the authors on request.
